# LPS-Inducible lncRNA TMC3-AS1 Negatively Regulates the Expression of IL-10

**DOI:** 10.3389/fimmu.2020.01418

**Published:** 2020-07-22

**Authors:** Mengling Ye, Minghong Xie, Jie Zhu, Chen Wang, Rui Zhou, Xiaoqing Li

**Affiliations:** ^1^Hubei Province Key Laboratory of Allergy and Immunology, Wuhan University School of Basic Medical Sciences, Wuhan University, Wuhan, China; ^2^Institute of Materials Research and Engineering, The Second Affiliated Hospital of Fujian Medical University, Quanzhou, China; ^3^Center for Stem Cell Research and Application, Union Hospital, Tongji Medical School, Huazhong University of Science and Technology, Wuhan, China

**Keywords:** NF-κB, TMC3-AS1, IL-10, innate immunity, TLR4

## Abstract

Long non-coding RNAs are essential regulators of the inflammatory response, especially for transcriptional regulation of inflammatory genes. It has been reported that the expression of transmembrane channel-like 3 (TMC3)–AS1 is increased following lipopolysaccharide stimulation. However, the potential function of TMC3-AS1 in immunity is largely unknown. Herein, we report a specific role for TMC3-AS1 in the regulation of inflammatory gene expression. TMC3-AS1 negatively regulates the expression of interleukin 10 (IL-10) in macrophage and intestinal epithelial cell lines. Mechanistically, TMC3-AS1 may interact with p65 in the nucleus, preventing p65 from binding to the κB consensus site within IL-10 promoter. These findings suggest that TMC3-AS1 may function as an important regulator in the innate immune response.

## Background

The innate immune system is the primary barrier to microbial pathogens (1). Macrophages and epithelial cells, with importance in the innate immune defense, are equipped with specific pathogen pattern recognition receptors (PRRs), functionally to recognize pathogen-associated molecular patterns, against pathogens causing infection (2, 3). The Toll-like receptors (TLRs) are classic PRRs. Response to specific pathogen recognition, TLRs recruit adaptor proteins and activate specific signaling cascades, such as nuclear factor kappa-B (NF-κB) signaling, to regulate the transcription of inflammatory genes, involved in antimicrobial host response (3–7). Inflammatory factors produced by immune cells mediate the inflammatory response, which is under the tight regulation to orchestrate the development of complex gene expression programs (1, 3, 4, 8). The aberrant expression of inflammatory genes can lead to tissue damage and chronic inflammatory diseases (5–8). The underlying innate immune response is driven by complex gene expression changes in each cell, and recent investigations have provided novel insights into the gene regulatory mechanisms underlying such gene transcriptional changes (3, 5, 6, 8). Currently, long non-coding RNAs (lncRNAs) have emerged as critical regulators of innate immune responses, particularly in NF-κB signaling (9). Importantly, the NF-κB signaling has been reported that be involved in transactivation or transrepression of lncRNA expression, such as HOTAIR (10) and FIRRE (11). Furthermore, LncRNAs also act as modulators, together with NF-κB signaling, to fine-tune inflammatory genes expression following specific immune stimuli (9, 11, 12). It has been reported that lncRNAs can interact with NF-κB subunits to suppress their DNA-binding activity, which results in the downregulation of NF-κB–responsive genes, such as MALAT1 (13). However, the potential molecular mechanisms of lncRNAs in the regulation of inflammatory genes expression are still largely unknown.

TMC3-AS1 is located antisense to the transmembrane channel-like 3 (TMC3) gene at the proximal region of human chromosome 15. Mouse Tmc3-as1 is located at the proximal region of chromosome 7. Although most lncRNAs appear to show poor evolutionary conservation across several species, especially in human and mouse, several lncRNAs have been shown functional conservatism in human and mouse, e.g., FIRRE (11), interleukin 7 (IL-7)–AS (12). Herein, we investigated the expression of TMC3-AS1 and its correlation with inflammatory factor expression in cells after lipopolysaccharide (LPS) treatment *in vitro* and *in vivo*. We found that LPS could induce TMC3-AS1 in intestinal epithelial cell line (SW480) and macrophage cell line (U937) *in vitro* and *in vivo*. Mechanically, TMC3-AS1 can negatively regulate the expression of IL-10 by interacting with p65 to inhibit its DNA-binding activity. Therefore, our data reveal a potential role of the LPS-responsive TMC3-AS1 in contributing to the transcriptional regulation of the inflammatory genes in LPS-stimulated cells.

## Materials and Methods

### Ethics Statement

The study protocol and drawing of the blood samples from human subjects were approved by the Research Ethics Committee of Wuhan University (Wuhan, Hubei, China). An informed consent was presented to all human subjects. The animal experiments were carried out in accordance with the recommendations of the Guide for the Care and Use of Laboratory Animals Monitoring Committee of Hubei Province, China. The protocol was approved by the Committee on the Ethics of Animal Experiments at the School of Basic Medical Sciences, Wuhan University.

### Cell Culture

SW480 cells, a human intestinal epithelial cell line, were cultured in Dulbecco modified eagle medium (DMEM) (Gibco, USA), with 10% fetal bovine serum (FBS) (Gibco, Rockville, MD, USA). U937 cells, a human macrophage cell line, were cultured in RPMI-1640, with 10% FBS. RAW264.7 cells, a murine macrophage cell line, were cultured in DMEM, with 10% FBS.

### Separation of Primary Mouse Macrophages and Human Monocytes

Primary mouse peritoneal macrophages (MPMs) and bone marrow–derived macrophages (BMDMs) were isolated from male mice (C57BL/6J, 4–6 weeks old) (Hubei Research Center of Laboratory Animals, Hubei, Wuhan, China). Mouse macrophages were collected and cultured as previously described (12, 14). After euthanasia, the mice were sprayed with 70% ethanol.

MPM: RPMI 1640 supplemented with 2% FBS was injected into the peritoneal cavity of mice. The cell suspension was collected into a 15-mL sterile polypropylene tube and centrifuged at 2,000 revolutions/min (rpm) for 5 min. The harvested cells were resuspended with RPMI 1640 medium, followed by centrifugation. Cells were cultured in RPMI 1640 medium supplemented with 10% FBS and incubated for 3 days, and the medium was then replaced with fresh medium. The non-adherent cells were removed by washing with phosphate-buffered saline (PBS). At 7 days, the adherent cells represented MPM.

Bone marrow–derived macrophages: the muscles connected the hind limb to the pelvis, and those connected the femur to the tibia were removed using fine scissors. Then, the femur was dissected using a strong scissor, by cutting through the tibia just below the knee joint and through the pelvic bone close to the hip joint. The bones were flushed with a disposable syringe filled with RPMI 1640. The cell suspension was collected into a 15-mL sterile polypropylene tube and centrifuged at 2,000 rpm for 5 min. Lyse red cells with lysis buffer (servicebio, Wuhan, Hubei, China). Fresh bone marrow cells were cultured in RPMI 1640 supplemented with 10% FBS and incubated for 3 days, and the medium was then replaced with fresh medium. At 7 days, the adherent cells represented BMDMs.

Isolation and culture of human peripheral blood mononuclear cells were reported previously (15). Briefly, human peripheral blood samples were collected from healthy volunteers. After having been collected in sodium citrate test tubes, the blood samples were mixed with Flight WG494 (servicebio) at a 1:1 ratio, followed by density gradient centrifugation. Human monocytes were seeded into a 10-cm dish at a density of 5 × 10^4^ cell/mL in 10 mL of RPMI 1640 medium (20% FBS) and incubated for 4 days, and the medium was then replaced with fresh medium. Primary mouse macrophages and human peripheral blood monocytes were stimulated with 1 μg/mL LPS.

### TMC3-AS1 Short Hairpin RNA Transfection and Reagents

Two short hairpin RNAs (shRNAs) targeting TMC3-AS1 (TMC3AS1 shRNA1 and TMC3-AS1 shRNA2) were both designed and synthesized by GenePharma (Shanghai, China), as well as negative control shRNA-NC. Cells were seeded at 0.2 × 10^6^ cell/well in a 24-well plate and incubated in the culture medium. When 50–70% confluence was reached, cells were transfected with shRNAs using Lipofectamine 2000 (Invitrogen, Carlsbad, CA, USA) according to the manufacturer's protocol. The shRNAs sequences were as follows: TMC3-AS1 shRNA1: 5′-ACTGTTCCCATTCTGATTTAA-3′, TMC3-AS1 shRNA2: 5′-GTGTGGAAGACATTCTAATAT-3′.

SW480, U937, 293T, or RAW264.7 cells were grown in 24-well plates. When 60–70% confluence was reached, cells were incubated with 1 μg/mL LPS, 100 ng/mL Pam3CSK4, and 100 ng/mL poly(I:C) (HMW), respectively. Lipopolysaccharide was purchased from Sigma (St. Louis, MO, USA). Pam3CSK4 and poly(I:C) (HMW) were purchased from Invivogen (San Diego, CA, USA).

### Plasmid Construction

The TMC3-AS1 expression vector (pcDNA3.1-TMC3-AS1) was constructed by cloning the reverse transcriptase–polymerase chain reaction (RT-PCR) product into *Kpn*I and *Not*I sites of the pcDNA3.1 vector (Invitrogen). The primers used for amplifying the full-length TMC3-AS1 transcript were as follows: F: 5′-ggtaccGGACGCCTTGGGCGGGCC-3′ and R: 5′-gcggccgcACATTTCATCTGTTATTTG-3′. Interleukin 10 luciferase reporter vectors were constructed by ligation of human IL-10 promoter region DNA fragment into *Kpn*I and *Mlu*l sites of PGL-Basic vector (Promega, Madison, WI, USA). The primers used for amplifying the promoter of IL-10 were F (−2,000/+200): 5′-ggtaccAGTGGACAGTAATTTCAAATCAAT-3′ and R (−2,000/+200): 5′-acgcgtCTCCTAGCCAATAAAGTATAGAGC-3′; F (−1,400/+200): 5′-ggtaccTCTGAGTTAGCTCCA-3′ and R (−1,400/+200): 5′-acgcgtCTCCTAGCCAATAAAGTATAGAGC-3′. The primers for PCR-mediated site-directed mutagenesis of IL-10 promoter, F: 5′-AAGGTGCAAGAA *AAAACCCT* CCAGGTCCTGGTCC-3′ and R: 5′-GGACCAGGACCTGG *AGGGTTTT*TTCTTGCACCTT-3′ (the underline represents the κB-binding sites).

### Quantitative Real-Time PCR

Total RNA was extracted from cells with Trizol (Life Technologies, Carlsbad, CA, USA) and then subjected to RT-PCR using a cDNA Reverse Transcription kit (TaKaRa, Dalian, Liaoning, China) according to the manufacturer's instructions. Real-time PCR analysis for gene expression was performed using TransStart™ SYBR Green qPCR Supermix (Vazyme, Nanjing, Jiangsu, China). The comparative CT (ΔΔCT) method was used to analyze the results, and GAPDH was used as an internal control for all the human and mice genes. The primers for real-time PCR are listed in Table S1.

### Western Blot

Western blots were performed as previously described (10). The following antibodies (Abs) were used for Western blot analysis: anti-p65 (D14E12; CST, Danvers, MA, USA), antitubulin (sc-166729; Santa Cruz, Dallas, TX, USA), and anti-H3 (4499S; CST).

### RNA Immunoprecipitation Assay

RNA immunoprecipitation (RIP) assay was performed as previously described (16). Briefly, 1 × 10^8^ cells for each immunoprecipitation were cross-linked with 1% formaldehyde for 10 min at 37°C and then glycine (125 mM) to quench the cross-linking reactions. Cells were lysed in RIP buffer [150 mM NaCl 50 mM Tris·HCl pH 7.5, 1 mM DTT, 1% Nonidet P-40 (NP-40), 0.5% sodium deoxycholate, 0.05% sodium dodecyl sulfate (SDS), 100 U/mL RNase, cocktail]. After that, cells were sonicated in an ice–water bath. The nuclei pellets were isolated and collected by centrifugation at 14,000 rpm for 10 min at 4°C. Isolated nuclei pellets were incubated with the specific Ab-coated A/G agarose beads (4 h, 4°C) and then washed with low salt buffer (50 mmol/L Tris·HCl pH 7.4, 150 mmol/L NaCl, 1 mmol/L MgCl_2_, 0.05% NP-40, 2 mmol/L EDTA, 1 mmol/L DTT, 100 U/mL RNase) and high salt buffer (50 mmol/L Tris·HCl pH7.4, 500 mmol/L NaCl, 1 mmol/L MgCl_2_, 0.05% NP-40, 2 mmol/L EDTA, 1 mmol/L DTT, 100 U/mL RNase). RNA fractions were extracted from the immunoprecipitates with Trizol (Life Technologies) and measured by real-time PCR. Anti-p65 (D14E12; CST) and immunoglobulin G (IgG) (ab125900; Abcam, Cambridge, MA, USA) were used for RIP.

### Chromatin Immunoprecipitation

Chromatin immunoprecipitation (ChIP) assay was performed as previously described (11). Briefly, 5 × 10^7^ cells for each immunoprecipitation were crosslinked with 1% formaldehyde. Glycine was added to quench the cross-linking reactions. Cells were lysed in lysis buffer (1% SDS, 10 mM EDTA, 50 mM Tris·HCl pH 8.1). After that, cells were sonicated in an ice–water bath. The chromatin fractions were incubated with the specific Ab-coated A/G agarose beads (4 h, 4°C) and then washed by wash buffers as follows: low salt buffer (0.1% SDS, 1% Triton X-100, 2 mM EDTA, 20 mM Tris·HCl pH 8.1, 150 mM NaCl), high salt buffer (0.1% SDS, 1% Triton X-100, 2 mM EDTA, 20 mM Tris·HCl pH 8.1, 500 mM NaCl), LiCl buffer (0.25 M LiCl, 1% NP-40, 1 mM EDTA, 10 mM Tris·HCl pH 8.1), and TE buffer. The complexes were eluted off the beads with elution buffer (1% SDS, 0.1 M NaHCO_3_). The percentage input method was used to normalize ChIP data. DNA fragments were purified using the Purification Kit (Tiangen, Beijing, China). Primers used for ChIP-qPCR were as follows: human IL-10, F: 5′-AGACACAGCCCAGAAAACC-3′, and R: 5′-TGCCAGGAAGCAGAGAGAA-3′. Anti-p65 (D14E12; CST) and IgG (ab125900; Abcam) were used for ChIP.

### Luciferase Reporter Assays

293T cells were grown in 24-well plates. When 60–70% confluence was reached, cells were cotransfected with *IL-10* promoter–reporter construct, pRL–TK–Renilla–luciferase, and TMC3-AS shRNA1/pcDNA3.1–TMC3-AS1, followed by luciferase assay detection (Promega) according to the manufacturer's protocol.

### Preparation of Nuclear and Cytosolic Fractions

Nuclear and cytosolic fractions from SW480 cells (about 5 × 10^6^ cells) were extracted using nuclear and cytoplasmic extraction reagents kit (Beyotime, Shanghai, China) according to the manufacturer's instructions. Briefly, SW480 cells were stimulated with LPS (1 μg/mL) for 8 h and then harvested and lysed in CEB-A, and CEB-B buffers to obtain cytosolic fractions. After centrifugation, the pellets were lysed in NEB buffer to obtain nuclear fractions. The total cellular extracts were for detecting p65 using tubulin as internal control, and the nuclear extracts were for detecting p65 protein using histone H3 as an internal control.

### Enzyme-Linked Immunosorbent Assay

SW480 cells were grown in six-well plates at 5 × 10^6^ cells per well. When cells reached 60–70% confluence, cells were transfected with TMC3-AS1 shRNA1 or pcDNA3.1-TMC3-AS1 for 24 h, followed by the addition of LPS (1 μg/mL) for 8 h. Cells were collected. And the level of IL-10 protein was measured using a human IL-10 enzyme-linked immunosorbent assay (ELISA) kit (SEA056Hu; USCN Life Science Inc., Shanghai, China) according to the manufacturer's instructions.

### Fluorescence *in situ* Hybridization

RNA–fluorescence *in situ* hybridization (FISH) was performed using the Fluorescent *in situ* Hybridization Kit (C10910; RiboBio, Guangzhou, Guangdong, China) as previously reported (11). The probes were labeled with Cy3. SW480 cells were cultured in 35-mm confocal dishes and stimulated with LPS (1 μg/mL) for 8 h. Leica LCS-SP8-STED (Medical Research Center for Structural Biology, Wuhan University) was used. The probes sequences were as follows: TMC3-AS1: 5′CY3-CACGCTTCTTAAACAAACGGTCGAC-3′.

### LPS Treatment *in vivo*

Female C57BL/6J normal (4–6 weeks old) mice were used for this study, approved by the Animal Care and Use Committee of Wuhan University. C57BL/6J mice were injected with LPS (15 mg/kg body weight) through intraperitoneal injection as previously described (11, 12). Animals from each group were sacrificed. The colon tissues (1-cm-long distal portions) were removed from mice and rinsed with PBS and then cut into several 3-mm-long pieces. Volumes of TRIzol reagent in sterile centrifuge tubes at 500-μL volumes were chilled on ice before homogenization of the colon tissues. The colon tissues were homogenized by Grinding Mill (servicebio). RNA was extracted from the homogenized colon tissues by using the TRIzol method (Life Technologies), and quantitative RT-PCR analysis was performed to detect the target genes expression.

### Statistical Analysis

All data were presented as means ± SD of three independent experiments. The analysis was performed using a Student *t*-test. *P* < 0.05 was considered significant.

## Results

### TMC3-AS1 Is Upregulated in LPS-Activated Human Cell Lines

**Figure 1:**
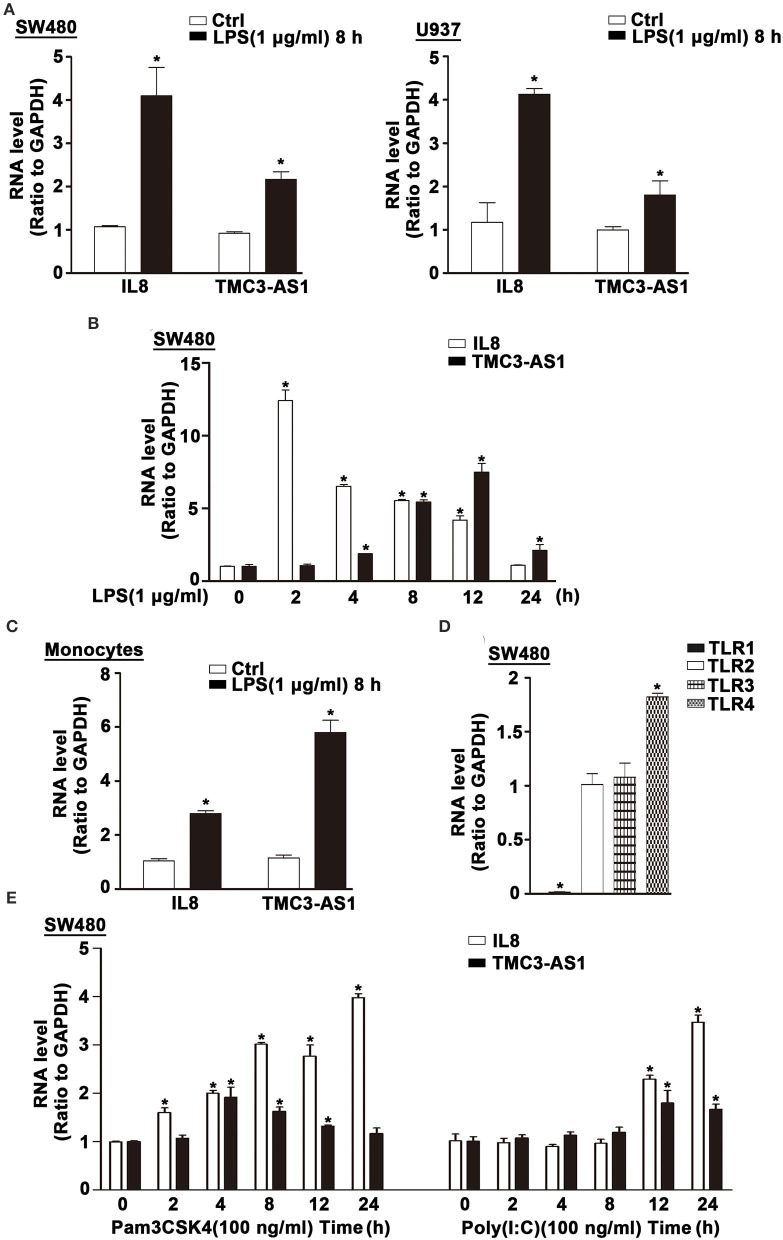
TMC3-AS1 is upregulated in LPS-activated human cell lines. **(A)** The expression of TMC3-AS1 in SW480 and U937 cells after exposure to LPS (1 μg/mL) for 8 h as assessed by using real-time PCR. IL-8 acted as a positive control. Data represent mean ± SEM, *n* = 3. *p < 0.05 vs. non-treated cells. **(B)** SW480 cells were treated with LPS (1 μg/mL) for up to 24 h, and real-time PCR detected the expression of TMC3-AS1 and IL-8. Data represent mean ± SEM, *n* = 3. *p < 0.05 vs. non-treated cells. **(C)** Monocytes were treated with LPS (1 μg/mL) for 8 h, and real-time PCR detected the expression of TMC3-AS1 and IL-8. Data represent mean ± SEM, *n* = 3. *p < 0.05 vs. non-treated cells. **(D)** The expression of TLR1, TLR2, TLR3, and TLR4 in SW480 cells as assessed by using real-time PCR. Data represent mean ± SEM, *n* = 3. *p < 0.05 vs. TLR2 expression level. **(E)** SW480 cells were treated with Pam3CSK4 (100 ng/mL) or poly(I:C) (100 ng/mL) for up to 24 h, and real-time PCR detected the expression of TMC3-AS1 and IL-8. Data represent mean ± SEM, *n* = 3. *p < 0.05 vs. non-treated cells.

Several lncRNAs were induced in human cell lines SW480 and U937 following LPS stimulation (11). The expression of TMC3-AS1 was upregulated by LPS stimulation in both cell lines (Figure 1A). Given the higher increase in TMC3-AS1 expression in both cell lines following LPS stimulation, we chose TMC3-AS1 for further study. We first performed the dynamic analyses of TMC3-AS1 expression in SW480 cells in response to LPS stimulation. Levels of TMC3-AS1 began to increase at 4 h and reached a peak level at 12 h following LPS stimulation. IL-8 acted as a positive control (Figure 1B). This variation tendency was similar to a typical late-primary response inflammatory gene (Figure 1B). We also found an increased expression of TMC3-AS1 in isolated human peripheral blood monocytes following LPS stimulation for 8 h (Figure 1C). Meanwhile, we measured the expression levels of TLR1, TLR2, TLR3, and TLR4 in SW480 cells by real-time PCR (Figure 1D) and performed the dynamic analyses of TMC3-AS1 expression in SW480 cells in response to Pam3CSK4 (100 ng/mL, an agonist of TLR1 and TLR2 signaling) and poly(I:C) (100 ng/mL, an agonist of TLR3 signaling) stimulation, respectively (Figure 1E). We found that there was a higher level of TLR4 than TLR1, TLR2, and TLR3 in SW480 cells (Figure 1D); the level of TMC3-AS1 reached a peak level at 4 h and increased at 8 h and 12 h following Pam3CSK4 stimulation (Figure 1E); when exposed to poly(I:C), the level of TMC3-AS1 reached a peak level at 12 h and increased at 24 h. These findings imply that TMC3-AS1 may play an important role in the innate immune response.

### TMC3-AS1 Regulates the Expression of Anti-Inflammatory Cytokine IL-10

**Figure 2:**
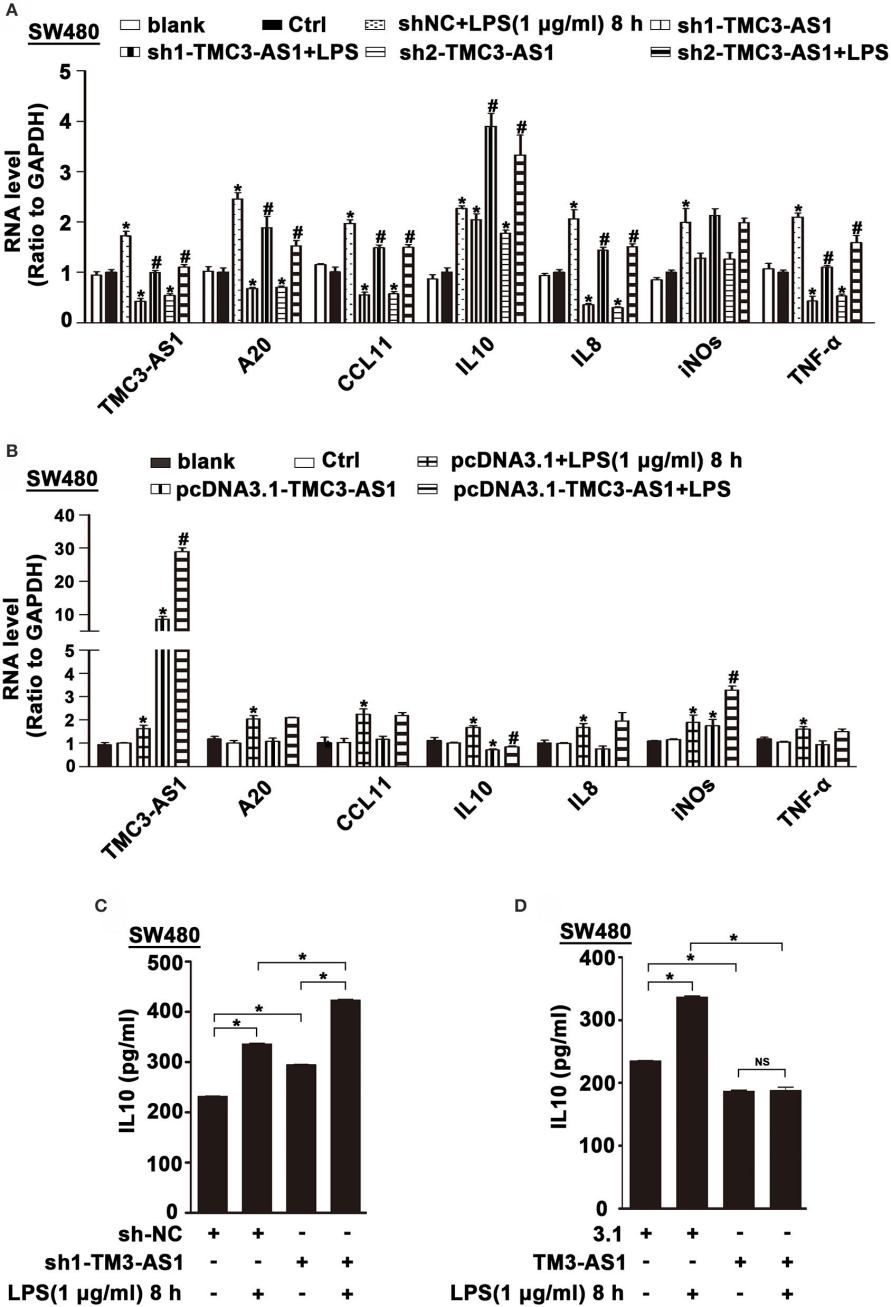
TMC3-AS1 regulates the expression of anti-inflammatory cytokine IL-10. **(A)** Real-time PCR analysis of TMC3-AS1 and selected inflammatory genes, A20, CCL11, IL-10, IL-8, iNOS, and TNF-α in SW480 cells transfected with the TMC3-AS1–shRNAs for 24 h and cultured for an additional 8 h in the presence or absence of LPS (1 μg/mL). Data represent mean ± SEM, *n* = 3. *p < 0.05 vs. control shRNA-treated cells, #*p* < 0.05 vs. LPS-stimulated cells. **(B)** Real-time PCR analysis of TMC3-AS1 and selected inflammatory genes, A20, CCL11, IL-10, IL-8, iNOS, and TNF-α in SW480 cells transfected with pcDNA3.1-TMC3-AS1 for 24 h and cultured for an additional 8 h in the presence or absence of LPS (1 μg/mL). Data represent mean ± SEM, *n* = 3. *p < 0.05 vs. control vector-treated cells, #*p* < 0.05 vs. LPS-stimulated cells. **(C)** ELISA analysis of IL-10 in SW480 cells transfected with the TMC3-AS1–shRNA1 for 24 h and cultured for an additional 8 h in the presence or absence of LPS (1 μg/mL). Data represent mean ± SEM, *n* = 3. *p < 0.05 vs. control cells. **(D)** ELISA analysis of IL-10 in SW480 cells transfected with the pcDNA3.1-TMC3-AS1 for 24 h and cultured for an additional 8 h in the presence or absence of LPS (1 μg/mL). Data represent mean ± SEM, *n* = 3. *p < 0.05 vs. control cells.

To identify potential genes regulated by TMC3-AS1, two shRNAs for TMC3-AS1 (TMC3-AS shRNA1, TMC3-AS shRNA2) were designed to target different regions of TMC3-AS1. Both shRNAs could effectively knock down the expression of TMC3-AS1 (Figure 2A). We measured the effects of gain- and loss-of-function of TMC3-AS1 on the expression of selected inflammatory genes in the non-stimulated and LPS-stimulated SW480 cells, including A20, CCL11, IL-10, IL-8, inducible nitric oxide synthase (iNOS), and tumor necrosis factor α (TNF-α). The knockdown of TMC3-AS1 decreased the expression of A20, CCL11, IL-8, and TNF-α and increased the expression of IL-10 in the non-stimulated and LPS-stimulated SW480 cells (Figure 2A). We transfected SW480 cells with pcDNA3.1-TMC3-AS1 and found overexpression of TMC3-AS1 increased the expression of iNOS and decreased the expression of IL-10 in the non-stimulated and LPS-stimulated SW480 cells (Figure 2B). The results reveal that TMC3-AS1 negatively regulated the expression of IL-10. Furthermore, we found that TMC3-AS1 knockdown increased the protein level of IL-10. And TMC3-AS1 overexpression decreased the protein level of IL-10 in both untreated and LPS-stimulated SW480 cells by using ELISA (Figures 2C,D). Moreover, LPS treatment failed to rescue the TMC3-AS1 overexpression–mediated downregulation of IL-10 in SW480 cells (Figure 2D).

### TMC3-AS1 Regulates the Expression of IL-10 Dependent on NF-κB

**Figure 3:**
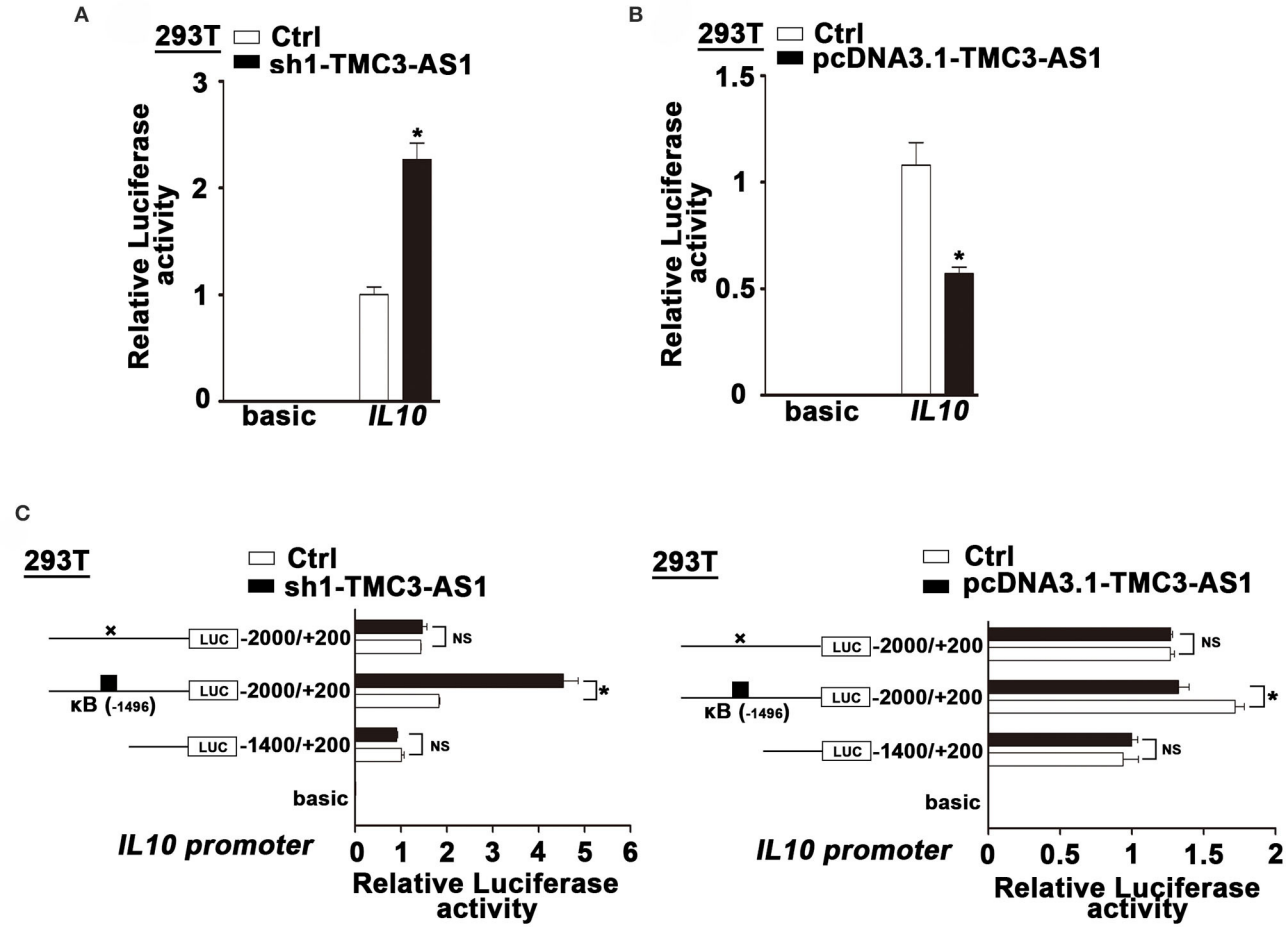
TMC3-AS1 regulates the expression of IL-10 dependent on NF-κB. **(A)** *IL-10* luciferase reporter activity in 293T cells upon TMC3-AS1 knockdown. Data represent mean ± SEM, *n* = 3. *p < 0.05 vs. control cells. **(B)** *IL-10* luciferase reporter activity in 293T cells upon TMC3-AS1 overexpression. Data represent mean ± SEM, *n* = 3. *p < 0.05 vs. control cells. **(C)** Luciferase reporter assays of *IL-10* promoter constructs containing truncated *IL-10* promoters or the κB–binding site mutated. Data represent mean ± SEM, n = 3. *p < 0.05 vs. control cells.

To clarify whether TMC3-AS1 regulates IL-10 expression at the transcriptional level, we constructed luciferase reporter plasmids containing *IL-10* gene promoters. TMC3-AS1 knockdown dramatically increased the *IL-10* promoter–luciferase reporter activity. Moreover, overexpression of TMC3-AS1 effectively inhibited the *IL*-*10* promoter–luciferase reporter activity in 293T cells (Figures 3A,B). The promoter regions of the *IL*-*10* gene contain functional κB-binding sites according to those previously described (17). To further investigate whether TMC3-AS1–mediated IL-10 expression is dependent on the κB-binding site in the IL-*10* gene promoter, we disrupted the site at −1,496 in the *IL*-*10* gene promoter by site-directed mutagenesis. As shown in Figure 3C, TMC3-AS1 knockdown or overexpression-mediated regulation of promoter activity of the IL-*10* gene was significantly impaired when the κB-binding site was disrupted. Similarly, there was no significant change of luciferase activity in TMC3-AS1 knockdown or overexpression cells transfected with constructs containing *IL*-*10* promoter without any κB-binding sites (Figure 3C). Taken together, these results demonstrate that TMC3-AS1 restrains the transcription of the IL-*10* gene through NF-κB–binding sites.

### TMC3-AS1 Interacts With p65 in the Nucleus

**Figure 4:**
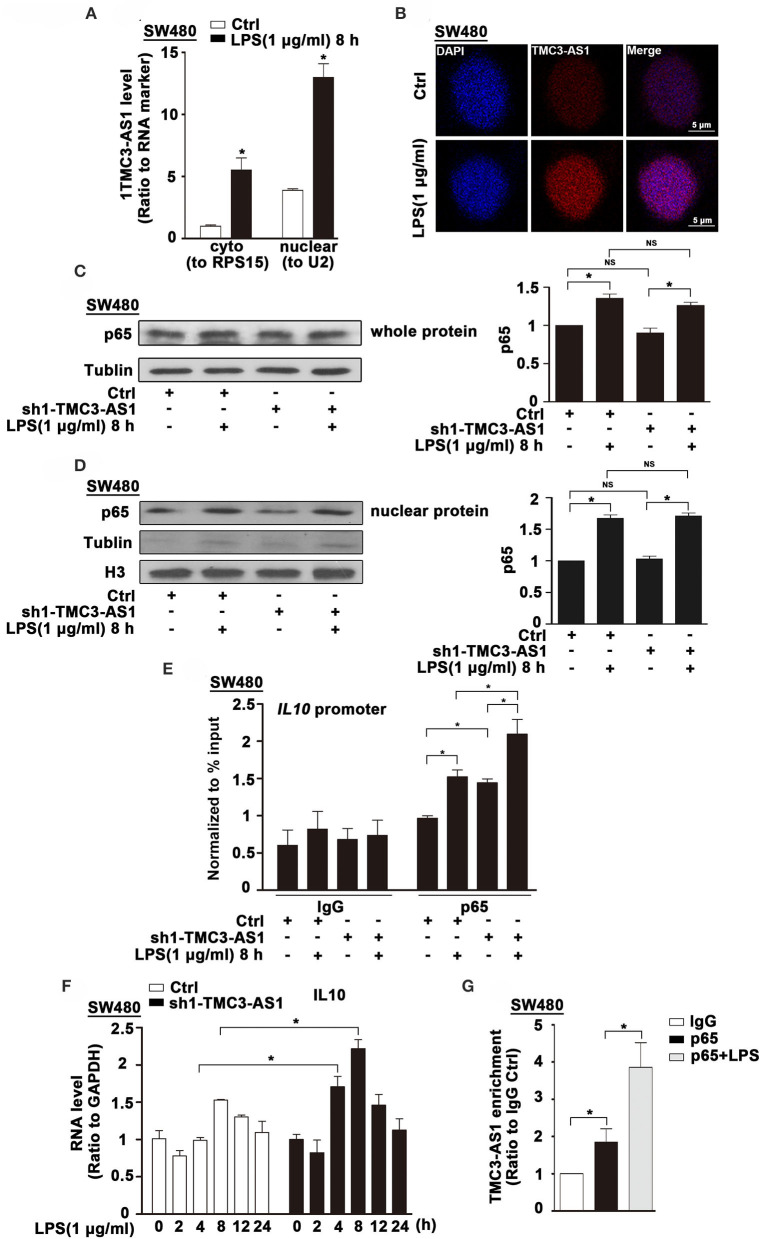
TMC3-AS1 interacts with p65 in the nucleus. **(A)** Nuclear vs. cytoplasmic distribution of TMC3-AS1 in SW480 cells following LPS stimulation (1 μg/mL) for 8 h. **(B)** RNA-FISH analysis of TMC3-AS1 in SW480 cells treated with LPS (1 μg/mL) for 8 h, using Cy3-labeled probes (red) for TMC3-AS1. The nuclei were stained with 4′,6-diamidino-2-phenylindole (DAPI, blue). Scale bar, 5 μm. **(C)** Western blot analysis of p65 in SW480 cells transfected with the TMC3-AS1–shRNA1 for 24 h and cultured for an additional 8 h in the presence or absence of LPS (1 μg/mL). Data represent mean ± SEM, *n* = 3. *p < 0.05 vs. control cells. **(D)** Western Blot analysis of nuclear distribution of p65 in control and TMC3-AS1 knockdown SW480 cells treated with LPS (1 μg/mL) for 8 h. **(E)** ChIP analysis of the recruitment of p65 to the *IL-10* promoter region in SW480 cells following LPS stimulation and its manipulation by TMC3-AS1 knockdown. Data represent mean ± SEM, *n* = 3. *p < 0.05 vs. control cells. **(F)** Real-time PCR detected the expression of IL-10 in control and TMC3-AS1 knockdown SW480 cells treated with LPS (1 μg/mL) for up to 24 h. Data represent mean ± SEM, *n* = 3. *p < 0.05 vs. non-treated cells. **(G)** RIP with anti-p65 revealed a physical association between TMC3-AS1 and p65 in LPS-stimulated and unstimulated SW480 cells. Data represent mean ± SEM, *n* = 3. *p < 0.05 vs. the control.

The subcellular localization of lncRNAs provides valuable clues to their molecular functions. We examined TMC3-AS1 localization by performing subcellular fractionation and real-time PCR in SW480 cells. This analysis revealed that TMC3-AS1 was predominantly nuclear in SW480 cells (Figure 4A). The nuclear localization of TMC3-AS1 was also confirmed by using RNA-FISH techniques (Figure 4B). Lipopolysaccharide-induced TLR4 signaling pathway predominantly converges on the activation of NF-κB signaling and its target genes, including IL-10. We wondered whether TMC3-AS1 inhibits IL-10 expression through regulating p65 (a transcription-activated subunit of NF-κB) abundance at the *IL*-*10* promoter. As shown in Figure 4C, there was no obvious difference in the protein level of p65 in TMC3-AS1 knockdown SW480 cells, compared with that in control cells. Furthermore, nuclear separation followed by Western blotting was performed to detect the effect of knockdown of TMC3-AS1 on p65 nuclear translocation in SW480 cells. The knockdown of TMC3-AS1 had no obvious effect on p65 nuclear translocation in untreated or LPS-treated SW480 cells (Figure 4D). To determine whether TMC3-AS1 could affect the binding of p65 to the *IL*-*10* promoter region, we performed ChIP analysis in LPS-activated SW480 cells. The knockdown of TMC3-AS1 remarkably increased the binding of p65 to the *IL*-*10* promoter region in both untreated and LPS-treated SW480 cells (Figure 4E). Besides, we also analyzed the kinetics of IL-10 mRNA expression in control and TMC3-AS1 knockdown SW480 cells following LPS stimulation. The expression of IL-10 increased at different time points in TMC3-AS1 knockdown SW480 cells following LPS stimulation compared with that of control (Figure 4F). Given the fact that TMC3-AS1 affects the binding of p65 to the *IL*-*10* promoter region, we speculated TMC3-AS1 interacts with p65 and prevents p65 from binding to κB-binding site in *IL*-*10* promoter. To test this possibility, we sought to determine whether intracellular TMC3-AS1 interacts with p65 by using formaldehyde cross-linking RNA-binding protein immunoprecipitation (RIP) in SW480 cells. We observed an obvious enrichment of TMC3-AS1 in the anti-p65 immunoprecipitates compared with the IgG control (Figure 4G). Moreover, LPS-treatment increased the physical interaction between TMC3-AS and p65 (Figure 4G), which may suggest that TMC3-AS1 physically interacts with p65 in the nucleus and thus prevents p65 from binding to its target sequences.

### Tmc3-as1 Is Induced by LPS in Mice

**Figure 5:**
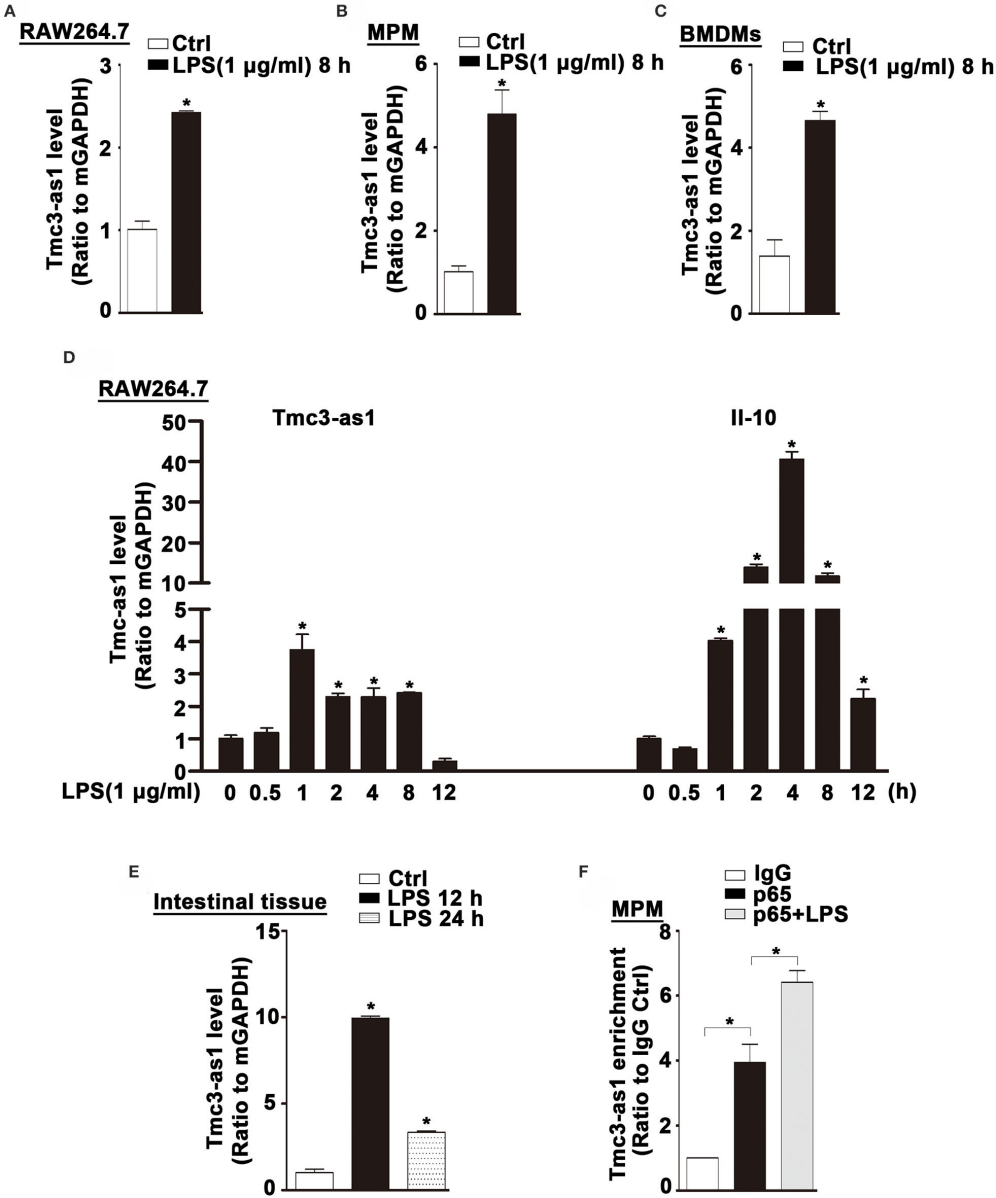
Tmc3-as1 be induced by LPS in mice. **(A)** RAW264.7 were treated with LPS (1 μg/mL) for 8 h, and real-time PCR detected the expression of Tmc3-as1. Data represent mean ± SEM, *n* = 3. *p < 0.05 vs. non-treated cells. **(B)** MPM were treated with LPS (1 μg/mL) for 8 h, and real-time PCR detected the expression of Tmc3-as1. Data represent mean ± SEM, *n* = 3. *p < 0.05 vs. non-treated cells. **(C)** BMDMs were treated with LPS (1 μg/mL) for 8 h, and real-time PCR detected the expression of Tmc3-as1. Data represent mean ± SEM, *n* = 3. *p < 0.05 vs. non-treated cells. **(D)** RAW264.7 were treated with LPS (1 μg/mL) for up to 12 h, and real-time PCR detected the expression of Tmc3-as1 and IL-10. Data represent mean ± SEM, *n* = 3. *p < 0.05 vs. non-treated cells. **(E)** Induction of Tmc3-as1t in intestine tissue isolated after LPS peritoneal injection (12 and 24 h), followed by real-time PCR. Data represent mean ± SEM, *n* = 3. *p < 0.05 vs. control group. **(F)** RIP with anti-p65 revealed a physical association between Tmc3-as1 and p65 in LPS-stimulated and unstimulated MPM. Data represent mean ± SEM, *n* = 3. *p < 0.05 vs. the control.

We found an increased expression of Tmcs-as1 in mouse macrophage cell line (RAW264.7), MPM, and BMDMs treated with LPS for 8 h (Figures 5A–C). To investigate the association of Tmcs-as1 with IL-10 in mice, we investigated a dynamic analysis of Tmcs-as1 and IL-10 expression in RAW264.7 cells following LPS stimulation. The expression of Tmcs-as1 reached a peak level at 1 h and increased at 2 h, 4 h, and 8 h following LPS stimulation (Figure 5D). The variation tendency of IL-10 expression was different from the expression of Tmcs-as1 in RAW264.7 cells after LPS treatment (Figure 5D). When LPS (15 mg/kg body weight) was injected into the abdominal cavity of C57BL/6J mice, a significant increase in Tmcs-as1 was detected in the intestinal tissues isolated 12 h and 24 h after LPS injection (Figure 5E). These results suggest LPS regulates Tmc3-as1 expression in mouse macrophages *in vitro* and *in vivo*. Moreover, we observed an obvious enrichment of Tmc3-as1 in the anti-p65 immunoprecipitates compared with the lgG control in MPM detected by using RIP analysis (Figure 5F). What's more, LPS treatment increased the physical interaction between Tmc3-as1 and p65 (Figure 5F).

## Discussion

Similar to protein-coding genes, transcriptions of lncRNAs are also regulated by several intracellular signaling pathways, including the TLR signaling pathways (10–12). A group of lncRNA genes has been demonstrated that act as TLR-responsive genes in mouse macrophages (18). In this study, not only TLR4 signaling pathways but also TLR 1/2 and TLR3 signaling pathways were involved in transcriptional regulation of TMC3-AS1, which suggested that TMC3-AS1 may play essential roles in the innate immune system. The effect of TLR1/2 and TLR3 agonists on increased expression of TMC3-AS1 was less obvious than the TLR4 agonist, which suggests that there was a higher expression of TLR4 than TLR1, TLR2, and TLR3 in SW480 cells. However, the more intricate molecular mechanisms underlying TLR-dependent transcriptional regulation of TMC3-AS1 need further study.

Previous studies have reported that many lncRNAs act as critical regulators to fine-tune inflammatory genes expression, for example, lncRNA-COX2 (9), HOTAIR (10). We investigated the biological functions of TMC3-AS1 in human cell lines SW480 and U937 through gain-of-function or loss-of-function approaches. The results revealed that TMC3-AS1 negatively regulates the expression of IL-10. Besides negatively regulating the expression of IL-10 in both human and mouse cell lines, knockdown of TMC3-AS1 could also decrease the expression of A20, CCL11, IL-8, and TNF-α in SW480 cells. Our results imply that TMC3-AS1 exerts an important role in the innate immunity by regulating the expression of several inflammatory genes. Nevertheless, whether other molecules are also involved in TMC3-AS1–mediated regulation of inflammatory genes is unclear.

To prevent excessive inflammation and maintain tissue homeostasis, several NF-κB–responsive genes, such as A20, have been reported to negatively regulate NF-κB activity (13, 19–21). Our data suggest that LPS-induced upregulation of TMC3-AS1 and p65 may form a nuclear RNA-protein complex and induced downregulation of the binding of p65 to the promoter of IL-10, which attenuates the expression of IL-10. The identification of TMC3-AS1, together with recently identified lncRNAs including MALAT1, NKIL-A, and Lethe, as novel NF-κB–binding molecules and regulators, extends the layer of complexity to the control of the key inflammatory genes critical in immunity and inflammation (13, 22, 23). In this study, we demonstrate that IL-10 is a target of TMC3-AS1 in human cell lines SW480 and U937. Thus, the expression increase in IL-10 induced by LPS stimulation in TMC3-AS1 knockdown SW480 cells was more obvious than that in control SW480 cells. Compared with protein-coding genes, the majority of lncRNAs show lower evolutionary conservation across species (24, 25). Nevertheless, several lncRNAs, such as FIRRE and IL-7-AS, exhibit the functional conservation across species (11, 12). Our work revealed that LPS induces the expression of TMC3-AS1 in both human and mouse cell lines. Moreover, TMC3-AS1 regulates the expression of IL-10 through an NF-κB–dependent mechanism in these cell lines. There was a slight increase in IL-10 expression in SW480 cells at 8 h after LPS stimulation, whereas there was a significant increase in RAW264.7 cells. Part of the reason is that the expression level of TMC3-AS1 may be different between SW480 and RAW264.7 cells. High-level expression of TMC3-AS1 prevented the LPS-induced expression of IL-10 in SW480 cells. Meanwhile, there was a lower level of Tmc3-as1 to slightly decrease the LPS-induced expression of IL-10 in RAW264.7 cells. The different expression levels of TMC3-AS1 may explain the different sensitivity to LPS stimulation in different cell lines and organs. Our results imply that TMC3-AS1 is a novel regulator of the immune response.

In summary, we functionally characterized an LPS-inducible lncRNA named TMC3-AS1, which is functionally conserved between humans and mice. TMC3-AS1 inhibits the expression of IL-10 by interacting with the transcription factor p65. Therefore, transcriptional inhibition of inflammatory genes by TMC3-AS1 may represent a new mechanism in the regulatory networks for inflammatory and immune responses, relevant to the development of new therapeutic strategies aimed at blocking specific TMC3-AS1–mediated transcriptional regulation.

## Data Availability Statement

The original contributions presented in the study are included in the article/Supplementary Material.

## Ethics Statement

The studies involving human participants were reviewed and approved by Research Ethics Committee of Wuhan University. The patients/participants provided their written informed consent to participate in this study. The animal study was reviewed and approved by Research Ethics Committee of Wuhan University.

## Author Contributions

XL and RZ conceived the conception. MY, MX, and JZ designed and performed the experiments. MY and CW wrote the manuscript. All authors contributed to the article and approved the submitted version.

## Conflict of Interest

The authors declare that the research was conducted in the absence of any commercial or financial relationships that could be construed as a potential conflict of interest.
