# Supplementary figures and images for: LPS-Inducible lncRNA TMC3-AS1 Negatively Regulates the Expression of IL-10
Source: Front Immunol. 2020 Jul 22;11:1418. doi: 10.3389/fimmu.2020.01418 (PMC7387720; doi:10.3389/fimmu.2020.01418)

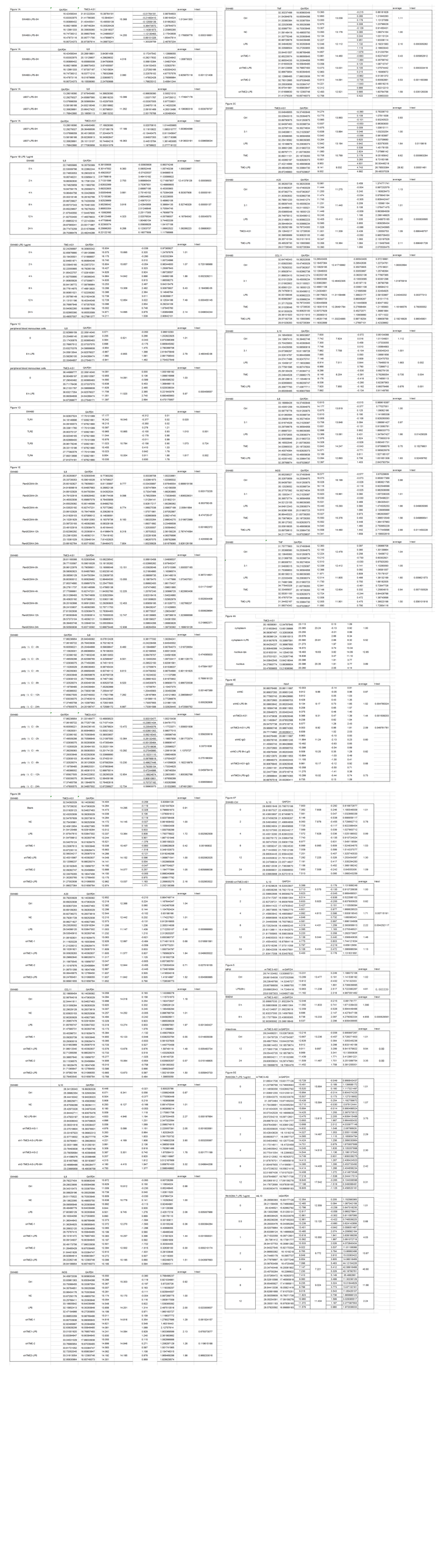

Supplement: Supplementary file 2 [file Image_5.tif]
