# Supplementary material for: LPS-Inducible lncRNA TMC3-AS1 Negatively Regulates the Expression of IL-10
Source: Front Immunol. 2020 Jul 22;11:1418. doi: 10.3389/fimmu.2020.01418 (PMC7387720; doi:10.3389/fimmu.2020.01418)
Supplement: Supplementary file 1 [file Data_Sheet_8.pdf]

Table S1. Primers for real-time PCR

Gene symbol	Sense primer (5'-3')	Antisense primer (5'-3')
TMC3-AS1	CTGAAGTCCCGAGACCACAACCC	TCTGCCCAAGACCTGGACACC
CCL11	CTTCTGTCCCAACCACCTGCT	GCTTTGGAGTTGGAGATTTTTGG
A20	TGTCACCAGCGTTCCAAGT	GACGCTGTGGGACTGACT
GAPDH	TGCACCACCAACTGCTTAGC	GGCATGGACTGTGGTCATGAG
IL10	TGGAGGACTTTAAGGGTTAC	TGATGTCTGGGTCTTGGTT
TLR1	GGAATGGCCCAAGGAAAAG	GTGAACTGCGACCCGAAGG
TLR2	CAGATGCCTCCCTCTTACCC	TTATTGCCACCAGCTTCCA
TLR3	ACTGATGCTCCGAAGGGTGG	ACCAGGGTTTGCGTGTTTCC
TLR4	TTAGACCTGTCCCTGAACCCT	AAACCAGCCAGACCTTGAATAC
IL8	GTGATGACTCAGGTTTGCCC	GGTGGTTTCTTCCTGGCTCT
TNF-α	GCCCTGGTATGAGCCCATCTAT	AGCCCCCAATTCTCTTTTTGAG
iNOS	GAGAGATTTTTCACGACACCCTT	GAAGGCAGCAGGCACACGCAAT
Tmc3-as1	AATAGATGGTTCACTCTGCCGTACTT	CTCTGAGAATGATTGGTTCCGCT
mGAPDH	ATGGTGAAGGTCGGTGTGAA	CGCTCCTGGAAGATGGTGAT
Il10	AAACAACTCCTTGGAAAACCTC	AGAACCCCTCCCATCATATAATA

Figure 1A	GAPDH	TMC3-AS1					average	t-test
SW480-LPS-0H	16.42458344	31.81222534	15.38764191	15.371	-0.01764191	0.98784603	1.0118584	
	15.83302975	31.41786385	15.5848341		-0.21483410	0.86164524		
	15.90886402	31.41445541	15.50559139		-0.13559139	0.91029661		
	16.89219856	31.89746284	15.00526428		0.36473572	1.28764573		
SW480-LPS-8H	16.12991333	30.35609360	14.22618027	14.255	1.14381973	2.20965285	2.176859778	0.000317953
	16.74738312	30.99607849	14.24869537		1.12130463	2.17543608		
	16.47873116	30.95771790	14.47898674		0.89101326	1.85447814		
	16.90724373	30.97397614	14.06673241		1.30326759	2.46787204		

Figure 1A	GAPDH	IL8					average	t-test
SW480-LPS-0H	16.42458344	20.26819801	3.84361458	4.016	0.17247542	1.12699055	1.00673023	
	15.83302975	20.13129807	4.29826832		-0.28217832	0.82234842		
	15.90886402	19.85680008	3.94793606		0.06815394	1.04837434		
	16.89219856	20.86675453	3.97455597		0.04153403	1.02920761		
SW480-LPS-8H	16.12991333	17.87307167	1.74315834	2.080	2.27293166	4.83304243	3.925675118	0.001121408
	16.74738312	18.53771210	1.79032898		2.22576102	4.67757576		
	16.47873116	19.01679688	2.53806572		1.47802428	2.78566984		
	16.90724373	19.15508060	2.24783688		1.76825312	3.40641244		

Figure 1A	GAPDH	IL8				average		t-test
U937-LPS-0H	13.28016090	27.67945480	14.39929390	13.399	-0.99939390	0.50021010	1.174947178	
	12.29279327	24.65897560	12.36618233		1.03371767	2.04729312		
	13.07698059	26.50995064	13.43297005		-0.03307005	0.97733831		
U937-LPS-8H	13.58198166	24.93218048	11.35019882	11.352	2.04970118	4.14020206	4.136080319	0.003079737
	12.35628891	23.66427422	11.30798531		2.09191469	4.26313486		
	11.76842880	23.16656113	11.39813232		2.00176768	4.00490404		

Figure 1A	GAPDH	TMC3-AS1					average	t-test
U937-LPS-0H	13.28016090	30.44945480	17.16929390	17.189	0.02070610	1.014455865	1.003604096	
	12.29279327	29.36468506	17.07189178		0.11810822	1.085310777		
	13.07698059	30.40138535	17.32440475		-0.13440475	0.911045647		
U937-LPS-8H	13.58198166	29.92260813	16.34062647	16.363	0.84937353	1.801718386	1.813633191	0.039659034
	12.35628891	29.10113107	16.74484216		0.44515784	1.361463065		
	11.76842880	27.77083958	16.00241078		1.18758922	2.277718122		

#DIV/0!

Figure 1B LPS 1μg/ml

SW480	IL8	GAPDH					average	t-test
0 h	22.74855995	16.35750389	6.39105606	6.383	-0.00905606	0.99374248	1.00033667	
	22.53009796	16.22862244	6.30147552		0.08052448	1.05740238		
	22.74653053	16.29032516	6.45620537		-0.07420537	0.94986516		
2 h	18.95746231	16.03956413	2.91789818	2.753	3.46410182	11.03566622	12.41378128	0.00009503
	18.88392830	16.17061234	2.71331596		3.66868404	12.71697857		
	18.74825859	16.11993790	2.62832069		3.75367931	13.48869905		
4 h	19.94759178	16.25556374	3.69202805	3.681	2.68997195	6.45300863	6.50307908	0.00000161
	19.96484756	16.33429909	3.63054848		2.75145152	6.73394306		
	20.00318146	16.28162766	3.72155380		2.66044620	6.32228555		
8 h	20.66729927	16.74200058	3.92529869	3.916	2.45670131	5.48960106	5.52746329	0.00000137
	20.65737343	16.70481300	3.95256042		2.42943958	5.38684135		
	20.65229607	16.78276253	3.86953354		2.51246646	5.70594745		
12 h	21.67540550	17.54457855	4.13082695	4.323	2.25117305	4.76069779	4.18784042	0.00045570
	21.82703400	17.46579933	4.36123466		2.02076534	4.05799007		
	21.69853210	17.22143364	4.47709846		1.90490154	3.74483339		
24 h	29.87565041	23.55171967	6.32393074	6.268	0.05806926	1.04107158	1.08266223	0.09880631
	29.77473259	23.51576996	6.25896263		0.12303737	1.08902522		
	29.70366478	23.48244286	6.22122192		0.16077808	1.11788988		

SW480 LPS 1μg/ml	TMC-AS1	GAPDH					average	t-test
0 h	32.24256897	16.30905342	15.934	15.895	-0.039	0.97365627	1.01	
	32.65676880	17.08135986	15.575		0.320	1.24797676		
	33.19435501	17.01888657	16.175		-0.280	0.82332364		
2 h	32.64651871	16.90694046	15.740	15.657	0.010	1.00724996	1.07	0.720198988
	32.03945160	16.23572731	15.804		-0.054	0.96344600		
	32.22000885	16.79295158	15.427		0.323	1.25087943		
4 h	31.85422707	17.02816391	14.826	14.842	0.924	1.89728557	1.88	0.002329211
	31.92755890	17.06399345	14.864		0.886	1.84860188		
	31.90805626	17.07306671	14.835		0.915	1.88558273		
8 h	30.84136772	17.58786964	13.253	13.309	2.497	5.64315479	5.43	2.18409E-05
	30.77614975	17.48813820	13.288		2.462	5.50975607		
	30.90851021	17.52209282	13.386		2.364	5.14646784		
12 h	31.05798149	18.31924438	12.739	12.854	3.011	8.06269917	7.49	0.000455145
	31.13101196	18.40344048	12.728		3.022	8.12534186		
	30.76997948	17.67357635	13.096		2.654	6.29234109		
24 h	33.94082260	18.93881798	15.002	14.695	0.748	1.67945760	2.14	0.049634244
	33.82665390	18.95553589	14.871		0.879	1.83894966		
	33.49057007	19.27981377	14.211		1.539	2.90642101		

Figure 1C

peripheral blood monouclear cells	IL8	GAPDH					average	t-test
0	22.92989159	22.35914040	0.571	0.521	-0.050	0.96610293	1.01	
	23.20468140	22.95010567	0.255		0.266	1.202822925		
	23.17430878	22.60995483	0.564		-0.043	0.970396369		
	22.76957512	22.07337570	0.696		-0.175	0.885645092		
8 h LPS	23.63270378	24.58699608	-0.954	-0.955	1.475	2.780399706	2.79	2.46044E-06
	24.05913544	24.92576027	-0.867		1.388	2.616475649		
	23.58290100	24.64289474	-1.060		1.581	2.991758543		
	22.33494857	23.27549171	-0.941		1.462	2.754027849		

peripheral blood monouclear cells	TMC3-AS1	GAPDH					average	t-test
0	36.44989777	22.35914040	14.091	14.091	0.000	1.000168192	1.03	
	36.90489197	22.95010567	13.955		0.136	1.099016998		
	37.29040482	22.60995483	14.680		-0.589	0.664596232		
	35.71178436	22.07337570	13.638		0.453	1.368496115		
8 h LPS	36.21331787	24.58699608	11.626	11.520	2.465	5.520038034	5.97	0.000459557
	36.63217354	24.92576027	11.706		2.385	5.221943076		
	35.99394608	24.64289474	11.351		2.740	6.680465663		
	34.67268677	23.27549171	11.397		2.694	6.470175957		

Figure 1D

SW480		GAPDH					average	t-test
TLR1	34.92837524	17.75101089	17.177	16.545	-6.312	0.01	0.020	
	34.18146896	17.93921661	16.242		-5.377	0.02		
	34.09165573	17.87621880	16.215		-5.350	0.02		
TLR2	28.33811760	17.75101089	10.587	10.865	0.278	1.21	1.010	0.001
	28.90375137	17.93921661	10.965		-0.100	0.93		
	28.92046165	17.87621880	11.044		-0.179	0.88		
TLR3	28.62689400	17.75101089	10.876	10.784	-0.011	0.99	1.073	0.724
	28.96178246	17.93921661	11.023		-0.158	0.90		
	28.33115196	17.87621880	10.455		0.410	1.33		
TLR4	27.77392578	17.75101089	10.023	10.004	0.842	1.79	1.817	0.002
	27.89313698	17.93921661	9.954		0.911	1.88		
	27.91074181	17.87621880	10.035		0.830	1.78		

Figure 1E

SW480	IL8	GAPDH					average	t-test
Pam3CSK4-0h	25.30293837	15.52930546	9.77363292	9.777	0.00336708	1.00233661	0.999919156	
	25.33726353	15.59018326	9.74708027		0.02991973	1.02095532		
	25.60192827	15.79056931	9.81135897		-0.03435897	0.97646554		
Pam3CSK4-2h	24.91809819	15.64857683	9.26952136	9.098	0.50747864	1.42156359	1.606529031	0.003173235
	24.89102928	15.84750394	9.04352534		0.73347466	1.66263865		
	24.79102918	15.80928482	8.98174436		0.79525564	1.73538485		
Pam3CSK4-4h	24.46302938	15.69897079	8.76405859	8.774	1.01294141	2.01802131	2.00641894	6.55375E-05
	24.65029382	15.80146599	8.84882783		0.92817217	1.90286362		
	24.53520192	15.82747231	8.70772962		1.06927038	2.09837189		
Pam3CSK4-8h	23.99102928	15.78474909	8.20628019	8.183	1.57071981	2.97052887	3.019672989	8.47472E-07
	24.01928103	15.87089012	8.14839091		1.62860909	3.09214740		
	24.10291829	15.90912093	8.19379736		1.58320264	2.99634270		
Pam3CSK4-12h	23.56720193	15.48392085	8.08328108	8.318	1.69371892	3.23489508	2.767474584	0.001662372
	23.48102918	15.02938475	8.45164443		1.32535557	2.50594642		
	23.62390292	15.20393814	8.41996478		1.35703522	2.56158226		
Pam3CSK4-24h	23.23810293	15.48392131	7.75418162	7.804	2.02281838	4.06376896	3.926126156	2.42055E-06
	23.15381029	15.33948104	7.81432925		1.96267075	3.89782886		
	23.68182764	15.83718392	7.84464372		1.93235628	3.81678065		

SW480	TMC3-AS1	GAPDH					average	t-test
Pam3CSK4-0h	28.61160088	15.52930546	13.08229542	13.151	0.06910458	1.04906537	1.000557165	
	28.77153587	15.59018326	13.18135262		-0.02995262	0.97945247		
	28.98122978	15.79056931	13.19066048		-0.03926048	0.97315366		
Pam3CSK4-2h	28.58092823	15.64857683	12.93235140	13.055	0.21904860	1.16396574	1.073407531	0.367214601
	29.08590129	15.84750394	13.23839735		-0.08699735	0.94148019		
	28.80393012	15.80928482	12.99464530		0.15675470	1.11477666		
Pam3CSK4-4h	27.95374680	15.69897079	12.25477600	12.225	0.89662400	1.86170437	1.922663406	0.011710497
	28.27811737	15.80146599	12.47665138		0.67474862	1.59631860		
	27.77099991	15.82747231	11.94352760		1.20787240	2.30996725		
Pam3CSK4-8h	28.31298493	15.78474909	12.52823584	12.453	0.62316416	1.54024960	1.62768237	0.002834408
	28.40920192	15.87089012	12.53831180		0.61308820	1.52952979		
	28.20192898	15.90912093	12.29280805		0.85859195	1.81326772		
Pam3CSK4-12h	28.24103837	15.48392085	12.75711753	12.747	0.39428247	1.31428893	1.323999598	0.000629694
	27.81302938	15.02938475	12.78364463		0.36775537	1.29034367		
	27.90393849	15.20393814	12.70000035		0.45139965	1.36736619		
Pam3CSK4-24h	28.57373104	15.48392131	13.08980973	12.938	0.06159027	1.04361550	1.169919126	0.219662371
	28.39483748	15.33948104	13.05535644		0.09604356	1.06883826		
	28.50593838	15.83718392	12.66875446		0.48264554	1.39730362		

SW480	GAPDH	IL8					average	t-test
poly（I：C）-0h	17.86236954	25.94049382	8.07812428	8.460	0.38177332	1.30294241	1.018729554	
	17.06185722	25.76403939	8.70218216		-0.24228456	0.84540552		
	16.60556221	25.20494868	8.59938647		-0.13948887	0.90784073		
poly（I：C）-2h	17.10928301	25.89086654	8.78158353	8.501	-0.32168593	0.80013430	0.981128173	0.474095833
	17.32390192	25.71903246	8.39513053		0.06476707	1.04591606		
	17.49509298	25.82098765	8.32589467		0.13400293	1.09733417		
poly（I：C）-4h	17.02938375	25.77450285	8.74511910	8.613	-0.28522150	0.82061561	0.901181625	0.475841507
	17.10293029	25.69038463	8.58745434		-0.12755674	0.91538037		
	17.39209383	25.89958495	8.50749112		-0.04759352	0.96754890		
poly（I：C）-8h	17.28303948	25.59059676	8.30755728	8.520	0.15234032	1.11137086	0.966720538	0.769918123
	17.02938103	25.77609485	8.74671382		-0.28681622	0.81970902		
	17.32528374	25.83049109	8.50520735		-0.04530975	0.96908173		
poly（I：C）-12h	17.18756485	24.54039484	7.35282999	7.262	1.10706761	2.15407370	2.296598437	0.001467389
	17.48386002	24.73930198	7.25544197		1.20445563	2.30450298		
	17.65927505	24.83749302	7.17821796		1.28167964	2.43121863		
poly（I：C）-24h	17.83039375	24.37463019	6.54423645	6.667	1.91566115	3.77286676	3.472590752	0.000292806
	17.37465758	24.10397563	6.72931805		1.73057955	3.31861105		
	17.47600575	24.20198747	6.72598172		1.73391588	3.32629445		

SW480	GAPDH	TMC3-AS1					average	t-test
poly（I：C）-0h	17.86236954	29.33146477	11.46909523	11.472	0.00310477	1.00215438	1.009001938	
	17.06185722	28.77257156	11.71071434		-0.23851434	0.84761772		
	16.60556221	27.84236145	11.23679924		0.23540076	1.17723371		
poly（I：C）-2h	17.10928301	28.60949603	11.50021302	11.364	-0.02801302	0.98077015	1.081772074	0.559200618
	17.32390192	28.70393849	11.38003657		0.09216343	1.06596749		
	17.49509298	28.70596849	11.21087550		0.26132450	1.19857858		
poly（I：C）-4h	17.02938375	28.50293839	11.47355464	11.292	-0.00135464	0.99906147	1.1370727	0.33731836
	17.10293029	28.30494193	11.20201164		0.27018836	1.20596527		
	17.39209383	28.59383503	11.20174120		0.27045880	1.20619136		
poly（I：C）-8h	17.28303948	28.62292838	11.33988890	11.230	0.13231110	1.09604809	1.192218979	0.275166304
	17.02938103	28.40391284	11.37453181		0.09766819	1.07004257		
	17.32528374	28.30120928	10.97592554		0.49627446	1.41056628		
poly（I：C）-12h	17.18756485	27.86620331	10.67863846	10.654	0.79356154	1.73334825	1.800382768	0.045979415
	17.48386002	28.48361015	10.99975014		0.47244986	1.38746354		
	17.65927505	27.94222832	10.28295326		1.18924674	2.28033651		
poly（I：C）-24h	17.83039375	28.39448573	10.56409199	10.734	0.90810801	1.87658289	1.674912801	0.009559843
	17.37465758	28.13948576	10.76482818		0.70737182	1.63282686		
	17.47600575	28.34857502	10.87256927		0.59963073	1.51532865		

Figure 2B

SW480	TMC3-AS1	GAPDH					average	t-test
Blank	32.54392029	18.14039382	14.404	14.295	-0.259	0.83594129	0.91	
	32.73728232	18.47363029	14.264		-0.119	0.921047824		
	33.01928123	18.54637463	14.473		-0.328	0.796691673		
	32.43202939	18.39283029	14.039		0.106	1.076091612		
NC	32.54787609	18.28373619	14.264	14.145	-0.119	0.920736408	1.00	
	32.75430981	18.58252938	14.172		-0.027	0.981608452		
	32.40912654	18.40927365	14.000		0.145	1.105843409		
LPS	31.54125468	18.02918294	13.512	13.364	0.633	1.550709299	1.72	0.002562509
	31.87547610	18.53847302	13.337		0.808	1.750778922		
	31.54709812	18.30293746	13.244		0.901	1.867151946		
shTMC-1	33.54076124	18.01928475	15.521	15.407	-1.376	0.385158323	0.42	0.00190832
	33.23067613	18.19303948	15.038		-0.893	0.538628829		
	33.87235110	18.20928474	15.663		-1.518	0.349153573		
shTMC-LPS	32.56034217	18.29387019	14.266	14.152	-0.121	0.919249266	1.00	0.002062025
	32.45310987	18.40392837	14.049		0.096	1.068671541		
	33.12098237	18.98028374	14.141		0.004	1.002985936		
shTMC-2	32.30192845	18.10956757	14.192	14.077	-1.014	0.495047585	0.54	0.002104791
	32.09282747	18.20456894	13.888		-0.710	0.611210601		
	32.33076355	18.18047456	14.150		-0.972	0.509696735		
shTMC2-LPS	31.30283765	18.12789450	13.175	13.037	0.003	1.002121096	1.11	0.004294307
	31.27302938	18.31068055	12.962		0.216	1.161227917		
	31.99027364	19.01656784	12.974		0.204	1.152122568		

SW480	A20	GAPDH					average	t-test
Ctrl	30.79203928	18.14039382	12.652	12.388	-0.210	0.864749714	1.04	
	30.69202938	18.47363029	12.218		0.224	1.167644347		
	30.92837204	18.54637463	12.382		0.060	1.042467636		
	30.69109283	18.39283029	12.298		0.144	1.104763424		
NC	30.82736272	18.28373619	12.544	12.442	-0.102	0.93198166	1.01	
	30.79281728	18.58252938	12.210		0.232	1.174227621		
	30.98271827	18.40927365	12.573		-0.131	0.912916855		
LPS	29.23526302	18.02918294	11.206	11.147	1.236	2.355314836	2.46	0.000668993
	29.54098120	18.53847302	11.003		1.439	2.712253107		
	29.53540910	18.30293746	11.232		1.210	2.312620207		
shTMC-1	31.08192831	18.01928475	13.063	12.997	-0.621	0.650380737	0.68	0.019561581
	31.11920228	18.19303948	12.926		-0.484	0.714911815		
	31.21028312	18.20928474	13.001		-0.559	0.678773251		
shTMC1-LPS	30.10291821	18.29387019	11.809	11.577	0.633	1.550734797	1.84	0.049935522
	30.00928283	18.40392837	11.605		0.837	1.785892874		
	30.29692840	18.98028374	11.317		1.125	2.181552728		
shTMC-2	31.15675545	18.10956757	13.047	12.944	-0.605	0.657385761	0.71	0.027018155
	31.10187676	18.20456894	12.897		-0.455	0.729354534		
	31.06701356	18.18047456	12.887		-0.445	0.734819084		
shTMC2-LPS	30.06445675	18.12789450	11.937	11.842	0.505	1.419554016	1.52	0.004560885
	30.24765451	18.31068055	11.937		0.505	1.41914897		
	30.66901655	19.01656784	11.652		0.790	1.728536773		

SW480	CCL11	GAPDH					average	t-test
Ctrl	32.19906454	18.14039382	14.059	14.018	0.193	1.143399278	1.18	
	32.56764018	18.47363029	14.094		0.158	1.115731673		
	32.54441811	18.54637463	13.998		0.254	1.192472937		
	32.31309284	18.39283029	13.920		0.332	1.258528124		
NC	32.29029128	18.28373619	14.007	14.252	0.245	1.185458294	1.01	
	32.83920153	18.58252938	14.257		-0.005	0.996766754		
	32.90283928	18.40927365	14.494		-0.242	0.845826911		
LPS	31.35857478	18.02918294	13.329	13.274	0.923	1.895539028	1.97	0.001340427
	31.85765747	18.53847302	13.319		0.933	1.908997951		
	31.47585731	18.30293746	13.173		1.079	2.11268862		
shTMC-1	33.40298731	18.01928475	15.384	15.123	-1.132	0.456376824	0.55	0.014835039
	33.09283739	18.19303948	14.900		-0.648	0.638253784		
	33.29383019	18.20928474	15.085		-0.833	0.561537605		
shTMC1-LPS	32.01902969	18.29387019	13.725	13.678	0.527	1.440770459	1.49	0.005483734
	31.98012040	18.40392837	13.576		0.676	1.59749119		
	32.71295056	18.98028374	13.733		0.519	1.433292625		
shTMC-2	33.38657640	18.10956757	15.277	15.064	-1.025	0.49140729	0.57	0.015148805
	33.11036575	18.20456894	14.906		-0.654	0.635605357		
	33.19028476	18.18047456	15.010		-0.758	0.591393302		
shTMC2-LPS	31.71365647	18.12789450	13.586	13.673	0.666	1.586929487	1.50	0.005843733
	31.97562184	18.31068055	13.665		0.587	1.502181054		
	32.78463540	19.01656784	13.768		0.484	1.39855059		

SW480	IL10	GAPDH					average	t-test
Ctrl	26.34128343	19.89282028	6.448	6.341	-0.321	0.80025786	0.87	
	25.39982354	19.32930294	6.071		0.056	1.039924938		
	26.44418342	19.94028323	6.504		-0.377	0.770090449		
NC	25.36625671	19.45829582	5.908	6.127	0.219	1.163958086	1.01	
	25.67558289	19.38284111	6.293		-0.166	0.891470044		
	25.42973328	19.24970245	6.180		-0.053	0.963909214		
NC-LPS-8H	23.94442711	18.93575478	5.009	4.946	1.118	2.170951758	2.27	0.000197664
	23.47933197	18.54612541	4.933		1.194	2.287534405		
	23.94308043	19.04709625	4.896		1.231	2.347322094		
shTMC3-AS-1	23.39331818	18.33538437	5.058	5.099	1.069	2.098074915	2.05	0.001933581
	23.37019804	18.39379501	4.976		1.151	2.220057391		
	23.33187523	18.06902313	5.263		0.864	1.82026425		
sh1-TMC3-AS-LPS-8H	22.57719002	18.28277779	4.294	4.169	1.833	3.561753732	3.90	0.003205567
	22.50760651	18.28626633	4.221		1.906	3.746802208		
	22.30311966	18.31250191	3.991		2.136	4.396581608		
shTMC3-AS-2	23.78493838	18.47585748	5.309	5.301	0.818	1.762861466	1.78	0.001771199
	23.79059584	18.40394848	5.387		0.740	1.67058413		
	23.41098376	18.20398496	5.207		0.920	1.892116867		
sh2-TMC3-AS-LPS-8H	22.61657685	18.30193857	4.315	4.415	1.812	3.512167669	3.32	0.049844208
	22.46989486	18.29028477	4.180		1.947	3.856761433		
	23.23995858	18.49038758	4.750		1.377	2.598049662		

SW480	IL8	GAPDH					average	t-test
Ctrl	29.78227404	18.90983049	10.872	10.812	-0.093	0.93728289	0.98	
	29.28023906	18.65094098	10.629		0.150	1.10934024		
	29.34310473	18.33387554	11.009		-0.230	0.85249945		
	30.08929196	19.35029388	10.739		0.040	1.02811520		
NC	29.51170522	18.70303948	10.809	10.779	-0.030	0.97964724	1.00	
	29.13622095	18.49855750	10.638		0.141	1.10292642		
	29.09595948	18.20393848	10.892		-0.113	0.92464981		
LPS	28.48486779	18.64039486	9.844	9.745	0.935	1.91126399	2.06	0.005007966
	27.80385129	18.30293848	9.501		1.278	2.42517219		
	28.16304056	18.27293855	9.890		0.889	1.85176110		
shTMC-1	31.36044943	18.98769486	12.373	12.270	-1.594	0.33130811	0.36	0.000394264
	31.36264925	18.99095845	12.372		-1.593	0.33155249		
	30.26932120	18.20394858	12.065		-1.286	0.40998055		
shTMC1-LPS	28.75767415	18.55849485	10.199	10.257	0.580	1.49466349	1.44	0.031550031
	29.15161472	18.76857493	10.383		0.396	1.31581824		
	30.09134937	19.90394845	10.187		0.592	1.50691609		
shTMC-2	30.49173756	17.99033938	12.501	12.502	-1.722	0.30304455	0.30	0.000210174
	31.26485429	18.87039485	12.394		-1.615	0.32636100		
	31.64401826	19.03384747	12.610		-1.831	0.28103646		
shTMC2-LPS	28.92994614	18.65803847	10.272	10.185	0.507	1.42118300	1.51	0.043597693
	29.22262146	19.12393746	10.099		0.680	1.60249073		
	28.84199954	18.65749373	10.185		0.594	1.50994311		

SW480	iNOS	GAPDH					average	t-test
Ctrl	34.45972039	18.90983049	15.550	15.303	-0.380	0.768496237	0.92	
	33.93961363	18.65094098	15.289		-0.119	0.921034661		
	33.70088455	18.33387554	15.367		-0.197	0.87235726		
	34.35764651	19.35029388	15.007		0.163	1.119339257		
NC	33.98404176	18.70303948	15.281	15.170	-0.111	0.925944557	1.00	
	33.67250176	18.49855750	15.174		-0.004	0.997269775		
	33.25768411	18.20393848	15.054		0.116	1.083917059		
LPS	33.18805942	18.64039486	14.548	14.201	0.622	1.539365092	2.00	0.023300637
	32.15852413	18.30293848	13.856		1.314	2.487013516		
	32.47194966	18.27293855	14.199		0.971	1.960183727		
shTMC-1	33.99853359	18.98769486	15.011	14.816	0.159	1.116637772	1.29	0.061524157
	33.80703936	18.99095845	14.816		0.354	1.278027686		
	32.82485667	18.20394858	14.621		0.549	1.46316443		
shTMC1-LPS	32.63928206	18.55849485	14.081	14.084	1.089	2.12757914	2.13	0.679373077
	33.01001920	18.76857493	14.241		0.929	1.903369599		
	33.83394947	19.90394845	13.930		1.240	2.361983662		
shTMC-2	33.04531029	17.99033938	15.055	14.846	0.115	1.082996888	1.26	0.119615188
	33.76980954	18.87039485	14.899		0.271	1.206297129		
	33.61721052	19.03384747	14.583		0.587	1.501741965		
shTMC2-LPS	32.72052045	18.65803847	14.062	14.185	1.108	2.154746315	1.98	0.969233016
	33.31613054	19.12393746	14.192		0.978	1.969469288		
	32.95830964	18.65749373	14.301		0.869	1.826629574		

SW480	TNF	GAPDH					average	t-test
Ctrl	32.30237488	18.90983049	13.393	13.039	-0.215	0.86181828	1.11	
	31.54394978	18.65094098	12.893		0.285	1.21840283		
	31.33380384	18.33387554	13.000		0.178	1.13137069		
	32.22029388	19.35029388	12.870		0.308	1.23799029		
NC	32.00997761	18.70303948	13.307	13.178	-0.129	0.91450431	1.00	
	31.59148419	18.49855750	13.093		0.085	1.06074164		
	31.33775248	18.20393848	13.134		0.044	1.03110124		
LPS	30.86726878	18.64039486	12.227	12.112	0.951	1.93338114	2.10	0.000305262
	30.34848282	18.30293848	12.046		1.132	2.19231583		
	30.33588504	18.27293855	12.063		1.115	2.16603043		
shTMC-1	33.84451557	18.98769486	14.857	14.460	-1.679	0.31233784	0.43	0.005852612
	32.85225574	18.99095845	13.861		-0.683	0.62274037		
	32.86554035	18.20394858	14.662		-1.484	0.35759742		
shTMC-LPS	31.61600028	18.55849485	13.058	13.031	0.120	1.08710747	1.11	0.000333419
	31.84123958	18.76857493	13.073		0.105	1.07574442		
	32.86675854	19.90394845	12.963		0.215	1.16085672		
shTMC-2	32.12986465	17.99033938	14.140	14.091	-0.962	0.51351372	0.53	0.001160368
	32.78312885	18.87039485	13.913		-0.735	0.60092881		
	33.25530856	19.03384747	14.221		-1.043	0.48516215		
shTMC2-LPS	30.97041881	18.65803847	12.312	12.521	0.866	1.82212213	1.59	0.035126339
	31.61698630	19.12393746	12.493		0.685	1.60764756		
	31.41376326	18.65749373	12.756		0.422	1.33953333		

Figure 2C

SW480	TMC3-AS1	GAPDH					average	t-test
Ctrl	33.64848955	19.37493848	14.274	13.993	-0.390	0.76336710	0.93	
	33.02943315	19.25394875	13.775		0.109	1.07811838		
	33.89790975	19.91293875	13.985		-0.101	0.93240523		
	32.94097463	19.00398734	13.937		-0.053	0.96393830		
3.1	33.22797104	19.35274634	13.875	13.884	0.009	1.00610111	1.00	
	33.04838611	19.21029387	13.838		0.046	1.03233254		
	33.30598095	19.36584958	13.940		-0.056	0.96183987		
LPS	32.25418582	18.99039385	13.264	13.184	0.620	1.53709680	1.64	0.0119818
	32.15168879	19.20938374	12.942		0.942	1.92078355		
	33.36601285	20.01983723	13.346		0.538	1.45178155		
TMC	32.89767171	21.83739283	11.060	10.789	2.824	7.07986142	8.62	0.000663284
	30.89311101	20.18729283	10.706		3.178	9.05165642		
	30.43060197	19.82928373	10.601		3.283	9.73163196		
TMC-LPS	27.42214999	18.46909838	8.953	9.032	4.931	30.50446218	28.92	0.00001481
	28.48049209	19.33984730	9.141		4.743	26.78503384		
	28.87246883	19.87029837	9.002		4.882	29.48337028		

SW480	A20	GAPDH					average	t-test
Ctrl	30.36283728	19.38202938	10.981	11.275	0.459	1.374771741	1.13	
	30.91862837	19.47463029	11.444		-0.004	0.997232579		
	30.87382774	19.67362837	11.200		0.240	1.180829473		
	31.30392832	19.82982738	11.474		-0.034	0.976640194		
3.1	30.78901233	19.04401274	11.745	11.440	-0.305	0.809442447	1.01	
	30.68097445	19.45009234	11.231		0.209	1.155981164		
	30.34469012	19.01100021	11.334		0.106	1.076471475		
	29.63885048	18.19000123	11.449		-0.009	0.993884939		
LPS	28.74975012	18.50456012	10.245	10.412	1.195	2.289146825	2.05	0.000830695
	29.31499013	18.90982223	10.405		1.035	2.048875185		
	29.59446434	19.00989234	10.585		0.855	1.809295434		
TMC3-AS1	30.29292189	18.76724300	11.526	11.339	-0.086	0.942340995	1.09	0.686446707
	29.12843217	18.12726530	11.001		0.439	1.35550753		
	30.39658989	18.90625100	11.490		-0.050	0.965709453		
TMC-LPS	28.35710154	18.01011910	10.347	10.364	1.093	2.133197526	2.11	0.696491728
	28.46528730	18.10900980	10.356		1.084	2.119497846		
	29.01726340	18.62726384	10.390		1.050	2.070530475		

SW480	CCL11	GAPDH					average	t-test
Ctrl	32.64746343	19.38202938	13.26543405	12.41718992	-0.80543405	0.57218991	1.08302694	
	31.63920393	19.47463029	12.16457364		0.29542636	1.22724761		
	31.78292032	19.67362837	12.10929195		0.35070805	1.27518631		
	31.95928741	19.82982738	12.12946003		0.33053997	1.25748394		
3.1	31.86603410	19.04401274	12.82202136	12.46402305	-0.36202136	0.77807365	1.01870918	
	32.01012329	19.45009234	12.56003095		-0.10003095	0.93301298		
	31.01902902	19.01100021	12.00802881		0.45197119	1.36790799		
	30.65601231	18.19000123	12.46601108		-0.00601108	0.99584210		
LPS	29.74760913	18.50456012	11.24304901	11.31158486	1.21695099	2.32454925	2.24040202	0.00377313
	30.00159046	18.90982223	11.09176823		1.36823177	2.58153967		
	30.60982967	19.00989234	11.59993733		0.86006267	1.81511715		
TMC	31.37170209	18.76724300	12.60445909	12.26491784	-0.14445909	0.90471852	1.16166576	0.76055552
	30.31028046	18.12726530	12.18301516		0.27698484	1.21165993		
	30.91353029	18.90625100	12.00727929		0.45272071	1.36861884		
TMC-LPS	29.36101920	18.01011910	11.35090010	11.33224885	1.10909990	2.15711022	2.19216828	0.86045801
	29.57182726	18.10900980	11.46281746		0.99718254	1.99609799		
	29.81029283	18.62726384	11.18302899		1.27697101	2.42329662		

SW480	IL10	GAPDH					average	t-test
Ctrl	24.19545930	16.36503857	7.830	7.624	-0.072	0.95104088	1.112	
	24.13697410	16.39482746	7.742		0.016	1.01104931		
	23.70425953	16.40393845	7.300		0.458	1.37333055		
3.1	24.43425056	16.68580818	7.748	7.758	0.010	1.00664683	1.001	
	24.67389297	16.81189728	7.862		-0.104	0.93045243		
	24.52914787	16.86445999	7.665		0.093	1.06681656		
LPS	24.07417488	16.92470741	7.149	7.011	0.609	1.52470753	1.683	0.002
	24.10459137	17.19032860	6.914		0.844	1.79469319		
	23.77581596	16.80747604	6.968		0.790	1.72866712		
TMC	25.29135429	17.00384712	8.288	8.204	-0.530	0.69279135	0.735	0.004
	25.24844528	17.09960175	8.149		-0.391	0.76268354		
	25.28128815	17.10548019	8.176		-0.418	0.74856113		
TMC-LPS	24.92959900	16.89209747	8.038	7.950	-0.280	0.82387563	0.876	0.001
	25.35677793	17.43677711	7.920		-0.162	0.89378466		
	24.85006485	16.95837593	7.892		-0.134	0.91149780		

SW480	IL8	GAPDH					average	t-test
Ctrl	33.18999426	19.37493848	13.815	13.819	-0.015	0.989618387	1.00	
	33.43051259	19.25394875	14.177		-0.377	0.77027001		
	33.58778778	19.91293875	13.675		0.125	1.09062186		
	32.61360594	19.00398734	13.610		0.190	1.141065338		
3.1	33.25859199	19.35274634	13.906	13.848	-0.106	0.929260093	0.97	
	32.91674529	19.21029387	13.706		0.094	1.066991427		
	33.29706678	19.36584958	13.931		-0.131	0.913060781		
LPS	31.88887331	18.99039385	12.898	13.081	0.902	1.868033773	1.66	0.01429326
	32.57872655	19.20938374	13.369		0.431	1.347847427		
	32.99559865	20.01983723	12.976		0.824	1.770600319		
TMC	36.16552546	21.83739283	14.328	14.247	-0.528	0.693451731	0.75	0.15279801
	34.02990033	20.18729283	13.843		-0.043	0.970898578		
	34.40074064	19.82928373	14.571		-0.771	0.585825578		
TMC-LPS	31.65822345	18.46909838	13.189	12.893	0.611	1.527185107	1.93	0.52409762
	32.43351452	19.33984730	13.094		0.706	1.631651308		
	32.26768874	19.87029837	12.397		1.403	2.643793754		

SW480	iNOS	GAPDH					average	t-test
Ctrl	35.95208527	19.37493848	16.577	16.199	-0.577	0.67028809	0.88	
	35.32670859	19.25394875	16.073		-0.073	0.950817369		
	35.94087581	19.91293875	16.028		-0.028	0.980821795		
	35.12029052	19.00398734	16.116		-0.116	0.922548599		
3.1	35.37757392	19.35274634	16.025	15.991	-0.025	0.982938068	1.01	
	35.13003417	19.21029387	15.920		0.080	1.057208335		
	35.39573774	19.36584958	16.030		-0.030	0.979496231		
LPS	34.52123155	18.99039385	15.531	15.293	0.469	1.384305431	1.66	0.049835095
	34.64912343	19.20938374	15.440		0.560	1.474535253		
	34.92961656	20.01983723	14.910		1.090	2.129065999		
TMC	36.86445323	21.83739283	15.027	15.376	0.973	1.962835957	1.56	0.049869541
	35.73679624	20.18729283	15.550		0.450	1.366510549		
	35.38127302	19.82928373	15.552		0.448	1.364157963		
TMC-LPS	33.00916835	18.46909838	14.540	14.480	1.460	2.750950229	2.88	0.011450228
	33.89759610	19.33984730	14.558		1.442	2.717445696		
	34.21177481	19.87029837	14.341		1.659	3.156932819		

SW480	TNF	GAPDH					average	t-test
Ctrl	31.75777663	19.37493848	12.383	12.479	0.097	1.06966708	1.04	
	31.35388580	19.25394875	12.100		0.380	1.30139864		
	32.13645083	19.91293875	12.224		0.256	1.19456712		
	32.21368755	19.00398734	13.210		-0.730	0.60302921		
3.1	31.86306701	19.35274634	12.510	12.412	-0.030	0.97920263	1.05	
	31.54938315	19.21029387	12.339		0.141	1.10260093		
	31.75389853	19.36584958	12.388		0.092	1.06581057		
LPS	30.66156313	18.99039385	11.671	11.805	0.809	1.75179107	1.60	0.009621573
	31.22333409	19.20938374	12.014		0.466	1.38132199		
	31.74981389	20.01983723	11.730		0.750	1.68182005		
TMC	34.77840328	21.83739283	12.941	12.604	-0.461	0.72647726	0.94	0.557150529
	32.33495937	20.18729283	12.148		0.332	1.25904815		
	32.55351100	19.82928373	12.724		-0.244	0.84426788		
TMC-LPS	30.47670734	18.46909838	12.008	11.901	0.472	1.38740698	1.50	0.556101918
	31.34452167	19.33984730	12.005		0.475	1.39023198		
	31.56074342	19.87029837	11.690		0.790	1.72854116		

Figure 4A

	TMC3-AS1						
	35.16056061	12.04797840	23.113		0.13	1.09	
cytoplasm	37.01653943	13.65139866	23.365	23.24	-0.13	0.92	1.00
	36.58387457	13.32933636	23.255		-0.01	0.99	
	36.08396124	15.50810013	20.576		2.66	6.34	
cytoplasm-+LPS	35.91907676	15.33897591	20.580	20.81	2.66	6.32	5.52
	36.64254227	15.36967850	21.273		1.97	3.91	
	32.80649399	14.23456454	18.572		3.74	13.34	
nucleus-lps	32.61833191	14.12540150	18.493	18.63	3.82	14.09	12.85
	33.07031021	14.23267746	18.838		3.47	11.10	
	34.03842545	13.64218044	20.396		1.91	3.77	
nucleus	34.27993774	13.88366604	20.396	20.35	1.91	3.77	3.89
	33.47859955	13.21836090	20.260		2.05	4.14	

Figure 4C

w hole protein

		GAPD	R	
nc	2274	2389	0.951862704	1
LPS	3060	2460	1.243902439	1.306808675
shTMC3-AS1-lps	2393	2042	1.171890304	1.231154765
shTMC3-AS1	2129	2177	0.977951309	1.02740795

		GAPD	R	
nc	1811	1856	0.97575431	1
LPS	2836	1983	1.430156329	1.465693068
shTMC3-AS1-lps	2365	1803	1.311702718	1.344296104
shTMC3-AS1	2213	2672	0.828218563	0.848798262

		GAPDH	R	
nc	2274	2494	0.911788292	1
LPS	3060	2586	1.183294664	1.297773479
shTMC3-AS1-lps	2793	2529	1.104389087	1.211234117
shTMC3-AS1	2029	2684	0.755961252	0.829097345

		GAPD	R	
nc	1125	3350	0.335820896	1
LPS	1789	3278	0.54575961	1.625150837
shTMC3-AS1-lps	1128	3370	0.334718101	0.996716123
shTMC3-AS1	1800	3298	0.545785324	1.625227411

		GAPD	R	
nc	1015	3290	0.308510638	1
LPS	1769	3214	0.55040448	1.784069695
shTMC3-AS1-lps	1032	3400	0.303529412	0.983853955
shTMC3-AS1	1812	3304	0.54842615	1.777657176

		GAPDH	R	
nc	1098	3288	0.333941606	1
LPS	1725	3197	0.539568345	1.615756575
shTMC3-AS1-lps	1233	3299	0.373749621	1.119206516
shTMC3-AS1	1987	3431	0.579131449	1.734229693

Figure 4E

SW480	p65	input					average	t-test
	30.88376480	20.88111687	10.003		-0.14	0.91		
shNC	30.86837283	20.95651245	9.912	9.86	-0.05	0.96	0.97	
	30.77583743	20.96428680	9.812		0.05	1.03		
	30.28372683	20.85595703	9.428		0.43	1.35		
shNC-LPS 8h	30.08603642	20.95203400	9.134	9.17	0.73	1.65	1.52	0.004785324
	30.16584748	20.95811653	9.208		0.65	1.57		
	30.20948373	20.83402443	9.375		0.48	1.40		
shTMC3-AS1	30.31473638	20.92625046	9.388	9.31	0.47	1.39	1.44	0.001508203
	30.11493847	20.87652588	9.238		0.62	1.54		
	29.54757736	20.97016716	8.577		1.28	2.43		
shTMC3-AS1-LPS	29.98990746	20.98674393	9.003	8.92	0.86	1.81	2.09	0.049781781
	29.77174862	20.93280411	8.839		1.02	2.03		
	30.84376480	20.88111687	9.963		-0.10	0.93		
shNC-IgG	32.94738474	20.95310974	11.994	11.24	-2.13	0.23	0.60	
	31.45583743	20.96428680	10.492		-0.63	0.65		
	31.25372683	20.85595703	10.398		-0.54	0.69		
shNC-LPS 8h-LgG	30.46584748	20.95811653	9.508	10.20	0.35	1.28	0.82	
	31.91736484	21.02335167	10.894		-1.03	0.49		
	31.98948373	20.83402443	11.155		-1.30	0.41		
shTMC3-AS1-LPS-IgG	30.72482837	20.74431038	9.981	10.17	-0.12	0.92	0.68	
	31.28473638	20.92625046	10.358		-0.50	0.71		
	32.24477364	20.97016716	11.275		-1.41	0.38		
shTMC3-AS1-IgG	31.23174862	20.93280411	10.299	10.02	-0.44	0.74	0.73	
	30.70972649	20.97485542	9.735		0.13	1.09		

Figure 4G

	TMC3-AS1						
	36.04515457	36.04515457	0		1		
	37.07191925	37.07191925	0		1		
IgG	36.51233292	36.51233292	0	0	1	1	
	35.41262323	36.04515457	-0.63253134		1.550282718		
	35.78774658	37.07191925	-1.28417267		2.435423503		0.038628011
p65	35.8237594	36.51233292	-0.68857352	-0.86842584	1.611689152	1.865798458	
	34.23982734	36.04515457	-1.80532723		3.49508424		
	34.75876961	37.07191925	-2.31314964		4.969668556		0.029846384
p65-LPS	34.82637754	36.51233292	-1.68595538	-1.93481075	3.21753397	3.894095589	

Figure 4F

SW480-Ctrl	IL10	GAPDH					
	28.69551849	20.79570198	7.900		-0.292	0.816872877	
0	29.81807327	22.45562553	7.362	7.608	0.246	1.185546509	1.01
	30.43603897	22.87469673	7.561		0.047	1.032869339	
	30.07459259	21.92908287	8.146		-0.538	0.688959117	
2	29.54934692	21.48664856	8.063	7.978	-0.455	0.729662713	0.78
	29.68924904	21.96469688	7.725		-0.117	0.922389404	
	30.52107393	22.95244217	7.569		0.039	1.027663712	
4	33.40013280	25.82802200	7.572	7.628	0.036	1.025188502	0.99
	32.08276172	24.33984756	7.743		-0.135	0.910724024	
	30.56747000	23.59091759	6.977		0.631	1.549118584	
8	30.10859247	23.10924530	6.999	6.995	0.609	1.524834675	1.53
	28.71443562	21.70512199	7.009		0.599	1.514337077	
	29.86064445	22.65944290	7.201		0.407	1.325740533	
12	29.04350632	21.76141548	7.282	7.225	0.326	1.253454097	1.30
	30.24798834	23.05714607	7.191		0.417	1.335294283	
	28.89212036	21.73639870	7.156		0.452	1.368199242	
24	29.90889931	22.05886650	7.850	7.506	-0.242	0.84555306	1.09
	32.03993988	24.52731514	7.513		0.095	1.068343259	

SW480-shTMC3-AS1	IL10	GAPDH					average	t-test
	35.91929626	16.52002907	19.399		0.179	1.131889249		
0	36.49508286	16.78217316	19.713	19.578	-0.135	0.910726836	1.00	
	36.58862686	16.96589279	19.623		-0.045	0.969468501		
	37.07417297	16.55991364	20.514		-0.936	0.522586102		
2	36.82729721	16.99397659	19.833	19.925	-0.255	0.837800925	0.82	
	36.86441422	17.43763542	19.427		0.151	1.110509094		
	35.36073685	16.75992775	18.601		0.977	1.968628523		
4	35.45883942	16.46658897	18.992	18.813	0.586	1.500818543	1.71	0.00715191
	35.66965866	16.82367897	18.846		0.732	1.66096343		
	35.34460068	16.76378822	18.581		0.997	1.996104908		
8	34.72525406	16.40665245	18.319	18.431	1.259	2.393958913	2.22	0.004252117
	34.81138611	16.41642570	18.395		1.183	2.270546521		
	35.81785965	16.59833908	19.220		0.358	1.282073907		
12	35.64935303	16.51160431	19.138	19.044	0.440	1.356840635	1.46	
	35.45544052	16.67994118	18.775		0.803	1.744121634		
	36.87016296	17.07311058	19.797		-0.219	0.85912956		
24	35.59042358	16.48437691	19.106	19.434	0.472	1.386986094	1.13	
	35.93417358	16.53457832	19.400		0.178	1.131631891		

Figure 5

MPM	mTMC	mGAPDH					average	t-test
	26.74133492	13.50995731	13.231		0.249	1.188072106		
Ctrl	26.86154938	13.57252598	13.289	13.477	0.191	1.141536192	1.02	
	28.23649788	14.3246727	13.912		-0.432	0.74132333		
	25.95786858	14.3688755	11.589		1.891	3.708939995		
LPS(8h)	25.68802643	14.7246418	10.963	11.238	2.517	5.722380267	4.81	0.003339
	25.81087303	14.64907169	11.162		2.318	4.987091462		

BMDM	mTMC	mGAPDH					average	t-test
	33.99987030	21.95226479	12.048		-0.215	0.86178177		
0 h	33.58800888	22.49601364	11.092	11.833	0.741	1.671339433	1.390	
	33.42134857	21.06229019	12.359		-0.526	0.694449466		
	32.83237350	23.14597893	9.686		2.147	4.427847156		
8 h	33.17377884	23.43808365	9.736	10.233	2.097	4.279092334	4.655	0.000629561
	32.90365082	23.36619949	9.537		2.296	4.909406665		

Intestines	mTMC	mGAPDH					average	t-test
	28.24468201	15.02873635	13.216		-0.006	0.995887267		
Ctrl	31.10479729	17.43754387	13.667	13.236	-0.457	0.728371603	1.01	
	28.46977654	15.64420795	12.826		0.384	1.305345236		
	29.09814453	19.18579674	9.912		3.298	9.833140156		
12	27.73951736	17.82840729	9.911	9.897	3.299	9.841579822	9.94	0.00
	29.05440735	19.18579674	9.869		3.341	10.13580936		
	28.59004211	17.15102386	11.439		1.771	3.412861221		
24	29.80655624	18.29747963	11.509	11.467	1.701	3.251089754	3.35	0.00
	30.15888878	18.7064476	11.452		1.758	3.381255001		

Figure 5E

RAW264.7 LPS 1ug/ml	mTMC3-AS	mGAPDH					
	31.66041708	15.93177128	15.729		-0.049	0.966843437	
0	31.23796768	15.74660683	15.491	15.684	0.189	1.139688172	1.01
	31.14658356	15.62662792	15.520		0.160	1.117321497	
	31.50661255	15.51255131	15.994		-0.314	0.804374225	
	31.50943375	16.00233078	15.507		0.173	1.127319952	
0.5	31.3973484	15.97165203	15.426	15.584	0.254	1.192759872	1.07
	31.75438881	16.04395294	15.710		-0.030	0.97912444	
	31.81404305	16.12026978	15.694		-0.014	0.990498524	
	30.57342529	16.14866829	14.425		1.255	2.387073512	
1	29.57254219	16.09712601	13.475	13.815	2.205	4.609415488	3.75
	29.86678123	16.17391777	13.693		1.987	3.964493432	
	29.97640991	16.30861282	13.668		2.012	4.033977148	
	30.55355835	15.92170334	14.632		1.048	2.06786928	
2	30.42843628	16.1018219	14.327	14.487	1.353	2.555110386	2.29
	30.66083717	16.0957737	14.565		1.115	2.165854794	
	30.54934692	16.12577248	14.424		1.256	2.389030964	
	31.17314911	16.4720459	14.701		0.979	1.970957666	
4	30.24605942	16.20081902	14.045	14.510	1.635	3.105357998	2.30
	30.77041054	16.2266922	14.544		1.136	2.198137543	
	30.91212082	16.16292572	14.749		0.931	1.906339275	
	31.87876701	17.46565819	14.413		1.267	2.406424523	
8	31.80497955	17.41995811	14.385	14.405	1.295	2.453733492	2.42
	32.47238232	18.05821419	14.414		1.266	2.404658234	
	32.01887436	17.61070251	14.408		1.272	2.414673546	
	33.97848907	16.76071739	17.218		-1.538	0.344417014	
12	36.03681812	17.09159279	18.945	17.542	-3.265	0.104008596	0.31
	34.17672699	16.97839165	17.198		-1.518	0.349088481	
	33.80383475	16.99889183	16.805		-1.125	0.458520161	

RAW264.7 LPS 1ug/ml	mIL10	mGAPDH					
	28.28590393	15.93177128	12.354		0.205	1.152580365	
0	28.21158409	15.74660683	12.465	12.559	0.094	1.067342158	1.01
	28.4246521	15.62662792	12.798		-0.239	0.847318235	
	28.13002586	15.51255131	12.617		-0.058	0.960278941	
	28.86338425	16.00233078	12.861		-0.302	0.811097095	
0.5	28.95236206	15.97165203	12.981	13.120	-0.422	0.746539225	0.68
	29.28300476	16.04395294	13.239		-0.680	0.624142856	
	29.52079964	16.12026978	13.401		-0.842	0.558051487	
	26.63399124	16.14866829	10.485		2.074	4.209582184	
1	26.71532059	16.09712601	10.618	10.550	1.941	3.839199206	4.03
	26.7361412	16.17391777	10.562		1.997	3.991072719	
	26.84157753	16.30861282	10.533		2.026	4.072840434	
	24.94327545	15.92170334	9.022		3.537	11.61106086	
2	24.86693382	16.1018219	8.765	8.772	3.794	13.86992496	13.87
	24.74465179	16.0957737	8.649		3.910	15.03363422	
	24.77978897	16.12577248	8.654		3.905	14.98018469	
	23.56763458	16.4720459	7.096		5.463	44.12154226	
4	23.34740448	16.20081902	7.147	7.221	5.412	42.58916488	40.60
	23.45700264	16.2266922	7.230		5.329	40.18790761	
	23.57265472	16.16292572	7.410		5.149	35.4882861	
	26.52610588	17.46565819	9.060		3.499	11.30236129	
8	26.65465927	17.41995811	9.235	9.024	3.324	10.01644629	11.66
	26.84413338	18.05821419	8.786		3.773	13.67132161	
	26.62691689	17.61070251	9.016		3.543	11.65426107	
	28.58359909	16.76071739	11.823		0.736	1.665688123	
12	28.05204391	17.09159279	10.960	11.434	1.599	3.028385517	2.24
	28.35001183	16.97839165	11.372		1.187	2.277387553	
	28.58020592	16.99889183	11.581		0.978	1.969304105	

MPM-RIP	TMC3-AS1							
	38.28373643	38.28373643	0.000		0.000	1		
IgG	37.09283723	37.09283723	0.000	0.000	0.000	1	1.00	
	38.49949345	38.49949345	0.000		0.000	1		
	36.2111698	38.28373643	-2.073		2.073	4.206343387		
p65	35.57398593	37.09283723	-1.519	-1.947	1.519	2.865627928	3.94	0.006308608
	36.25079346	38.49949345	-2.249		2.249	4.752544042		
	35.67716287	38.28373643	-2.607		2.607	6.090554402		
p65-LPS	34.51271973	37.09283723	-2.580	-2.673	2.580	5.979884037	6.40	0.021285512
	35.66726303	38.49949345	-2.832		2.832	7.121743236		
